# Medial septum regulates the hippocampal spatial representation

**DOI:** 10.3389/fnbeh.2015.00166

**Published:** 2015-06-30

**Authors:** Omar Mamad, Harold M. McNamara, Richard B. Reilly, Marian Tsanov

**Affiliations:** ^1^Trinity College Institute of Neuroscience, Trinity College DublinDublin, Ireland; ^2^School of Psychology, Trinity College DublinDublin, Ireland; ^3^Trinity Centre for Bioengineering, Trinity College DublinDublin, Ireland; ^4^School of Engineering, School of Medicine, Trinity College, University of DublinDublin, Ireland

**Keywords:** hippocampus, medial septum, optogenetics, theta rhythm, transgenic rat

## Abstract

The hippocampal circuitry undergoes attentional modulation by the cholinergic medial septum. However, it is unclear how septal activation regulates the spatial properties of hippocampal neurons. We investigated here what is the functional effect of selective-cholinergic and non-selective septal stimulation on septo-hippocampal system. We show for the first time selective activation of cholinergic cells and their differential network effect in medial septum of freely-behaving transgenic rats. Our data show that depolarization of cholinergic septal neurons evokes frequency-dependent response from the non-cholinergic septal neurons and hippocampal interneurons. Our findings provide vital evidence that cholinergic effect on septo-hippocampal axis is behavior-dependent. During the active behavioral state the activation of septal cholinergic projections is insufficient to evoke significant change in the spiking of the hippocampal neurons. The efficiency of septo-hippocampal processing during active exploration relates to the firing patterns of the non-cholinergic theta-bursting cells. Non-selective septal theta-burst stimulation resets the spiking of hippocampal theta cells, increases theta synchronization, entrains the spiking of hippocampal place cells, and tunes the spatial properties in a timing-dependent manner. The spatial properties are augmented only when the stimulation is applied in the periphery of the place field or 400–650 ms before the animals approached the center of the field. In summary, our data show that selective cholinergic activation triggers a robust network effect in the septo-hippocampal system during inactive behavioral state, whereas the non-cholinergic septal activation regulates hippocampal functional properties during explorative behavior. Together, our findings uncover fast septal modulation on hippocampal network and reveal how septal inputs up-regulate and down-regulate the encoding of spatial representation.

## Introduction

The projections from the medial septum to the hippocampus are proposed to have important roles in cognition by modulating the activity of episodic memory circuits (Winson, 1978; Oddie et al., 1996). Beyond its role as a pacemaker for hippocampal rhythmic oscillations (Dragoi et al., 1999), the function of the septal network remains underexplored. The precise spatial and temporal effect of septal activation on hippocampal neurons remains unclear making it difficult to define specific roles for septal activity in hippocampus-dependent behaviors. Hippocampal place cells encode the spatial representation (O'keefe, 1976) and their spiking is entrained by theta rhythm (O'keefe and Recce, 1993). The septo-hippocampal oscillatory regulation of the neuronal activity allows the precise timing and synchronization of the postsynaptic potentials arriving at hippocampal pyramidal cells (Freund and Gulyas, 1997). If medial septum exerts precise temporal control of hippocampal function then it is crucial to understand how medial septum shapes the hippocampal spatial representation.

Lesions of medial septum impair hippocampal theta and rhythmic discharge of hippocampal interneurons (Rawlins et al., 1979; Buzsaki et al., 1983). Concurrently, septal inactivation decreases the spiking of hippocampal pyramidal neurons and reduces their ability to fire in trains (Leutgeb and Mizumori, 1999; Koenig et al., 2011). Selective cholinergic septo-hippocampal lesions are also known to attenuate hippocampal theta (Lee et al., 1994; Yoder and Pang, 2005). Although the pharmacological effects of exogenous application of cholinergic agonists have been extensively studied in hippocampal neurons, much less is known about the system effects of septal cholinergic activation on septo-hippocampal circuitry. We investigated, here, if stimulation of the cholinergic septal neurons will trigger a network effect in medial septum or if the response will be restricted to the hippocampal formation. Another possible outcome would be concurrent stimulation-induced changes in the septal network and the hippocampal network. We applied novel approach including optogenetic stimulation in the medial septum of choline acetyltransferase (Chat)::Cre rat line (Witten et al., 2011). This method allowed us to evaluate for the first time the functional network connectivity in medial septum. The current understanding of intra-septal connectivity between medial septal cholinergic and non-cholinergic neurons is based on anatomical (Leranth et al., 1992; Brauer et al., 1998) electrophysiological recordings *in vitro* (Sotty et al., 2003; Huh et al., 2010) and under anesthesia (Bassant et al., 2005; Simon et al., 2006). We investigated here if cholinergic neurons exert strong intra-septal network effect on septal bursting cells. We explored how the septal cholinergic activation modulates the hippocampal and septal networks.

Septal cholinergic neuromodulation is crucially involved in the shifting of behavioral states (Lee and Dan, 2012) but there is scarce information about the role of cholinergic processing during navigation. Novel findings have demonstrated that the selective septal cholinergic effect on hippocampal theta activity is stronger during recordings from mice under anesthesia compared to recordings from awake animals (Vandecasteele et al., 2014). However, it remains unclear if this result was not related to the anesthetic effect on the neuronal activity (Kaifosh et al., 2013). Therefore, we also included the behavioral state of the rats as a factor in our investigation of the septo-hippocampal dialog. We aimed to examine if selective activation of the septal cholinergic projections are sufficient enough to evoke changes in the neuronal spiking and oscillatory patterns of hippocampal formation during exploratory behavior. We hypothesized that if the selective cholinergic activation is not able to trigger a potent network response during active behavior then a non-selective stimulation of septo-hippocampal projections should reveal the effect of septal activity on hippocampal place fields. Our findings show that the spiking of hippocampal place cells and their spatial representation is up-regulated or down-regulated, depending on the precise timing of non-selective septal activation.

## Materials and methods

### Surgical implantation of electrodes

The surgical implantation and the single-unit recordings were performed as previously described (Tsanov et al., 2011a). Eight tetrodes were implanted in medial septum (+0.5 AP, 1.1 ML, angle 10° medially and 5.5 mm dorsoventral to dura) or hippocampus (−3.8 AP, 2.3 ML, and 1.8 mm dorsoventral to dura) of four male (250–350 g) Lister-Hooded rats (Harlan, UK) and seven male (250–350 g) Lister-Hooded Chat::Cre rats (Rat Resource & Research Center P40OD011062, US). For simultaneous LFP recordings, in parallel with the tetrodes implantation, a single electrode was implanted in the medial septum or hippocampus. Experiments were conducted in accordance with European Community directive, 86/609/EC, and Cruelty to Animals Act, 1876, and followed Bioresources Ethics Committee, Trinity College Dublin, Ireland, and international guidelines of good practice.

### Recording techniques

Subjects were connected, via a 32 channel headstage (Axona Ltd.), to a recording system, which also allowed for animal position tracking. The recordings took place in rectangle-shaped linear track (80 × 80 × 9.5 cm and wide) situated in the center of a room with multiple background cues available. Signals were amplified (× 10,000–30,000) and band-pass filtered between 380 Hz and 6 kHz for single-unit detection. To maximize cell separation, only waveforms of sufficient amplitude (at least three times noise threshold) were acquired. Candidate waveforms were discriminated off-line using graphical cluster-cutting software (Axona Ltd.), which allows waveform separation based on multiple features including spike amplitude, spike duration, maximum and minimum spike voltage, and the time of occurrence of maximum and minimum spike voltages. Autocorrelation histograms were calculated for each unit, and the unit was removed from further analysis if the histogram presented spiking within the first 2 ms (refractory period), inconsistent with good unit isolation.

### Recording sessions

For the inactive (immobile) recording session pellets were positioned in the middle of the square arena where the rats consumed the pellets throughout the recordings session. For the active (mobile) recording session the rats were placed in the open field and 20 mg food pellets (TestDiet, Formula 5TUL) were thrown in every 20 s to random locations within the open field (pellet-chasing task); in this way, animals locomoted continuously, allowing for complete sampling of the environment. A recording session was considered as inactive if the animal locomoted less than 14 m per 12 min (with average velocity of <2 cm/s) and active if the animal locomoted more than 72 m per 12 min (average velocity of >10 cm/s). The chosen duration of 12 min allowed the rats to explore evenly the arena in two subsequent recordings (baseline and stimulation sessions). Baseline recordings >15 min result in insufficient exploration of the subsequent TBS session, while recordings < 10 min reduced the sampling of the explored environment. The rats were habituated to the square arena before the recordings.

### Hippocampal units identification and spatial firing analysis

Single hippocampal pyramidal cells and interneurons were identified using spike shape and firing frequency characteristics (Wilson and Mcnaughton, 1993; Csicsvari et al., 1999). Firing rate maps allow for visual inspection of neurons preferred areas of firing (i.e., place fields). They were constructed by normalizing the number of spikes which occurred in specific pixel coordinates by the total trial time the animal spent in that coordinate. This produced maps depicting the place fields of each cell and quantified in Hertz. The pixel map is converted into a 32 × 32 array of square bins 2 cm on a side. We computed place field size as the region of the arena in which the firing rate of the place cell was 20% or greater of the maximum firing frequency (Hollup et al., 2001; Brun et al., 2002). We used multiple indices to analyze the spatial properties of the hippocampal place cell firing (i.e., place field size, spatial selectivity, spatial coherence and spatial specificity information content). The spatial information of firing field (ratio of maximal signal to noise) was calculated by dividing the firing rate of the cell in the bin with the maximum average rate by its mean firing over the entire apparatus (Skaggs et al., 1996). Spatial coherence consists of a spatial autocorrelation of the place field map and measures the extent to which the firing rate in a particular bin is predicted by the average rate of the eight surrounding bins. Thus, high positive values result if the rate for each bin could be better predicted given the firing frequency of the neighboring location (Muller and Kubie, 1989; Quirk et al., 1990; Sharp and Green, 1994). With each spatial autocorrelation performed on the place field map, a *p*-value is calculated, indicating whether the correlation is significant or not. Place field activity is not considered to be spatially coherent if the *p*-value is greater than 0.001. The spatial information content (or spatial specificity) is expressed in bits per spike (Skaggs et al., 1993) and is calculated as follows:

(1) $$I=\sum_{i}P_{i}\left(\lambda_{i}/\lambda \right)log_{2}(\lambda_{i}/\lambda )$$

Where λ_i_ is the mean firing rate in bin *i*, λ is the overall mean firing rate and *P_i_* is the occupancy probability of bin *i*. The spatial specificity index is a measure of the amount of information relative to the location of the animal conveyed by a single action potential emitted by a single place cell.

### Measurement of local field activity

The local field potential (LFP) recordings were performed as previously described (Tsanov et al., 2011b). The LFP was sampled at 250 Hz and stored for off-line analysis. LFP signal frequency analysis was implemented with MATLAB's Signal Processing Toolbox (MATLAB, Natick, MA) where the power was calculated using the short-time Fourier transform of the signal (Hanning window of 2 s, with overlap of 1 s) and interpolated into color-coded power spectrograms. Information was displayed as the magnitude of the time-dependent Fourier transform vs. time in a color gradient graph with the maximum corresponding to 0 dB. Theta rhythm parameters were evaluated by off-line band-pass filtering of the signal in the range of 5–12 Hz.

### Phase-locking value

To evaluate the effect of optogenetic septal stimulation we compared the hippocampal local field oscillations of a single electrode between multiple trials. Phase-locking statistics allow investigation of the phase covariance between separate signals and allows direct quantification of frequency-specific synchronization (i.e., transient phase-locking) between local field potentials (Lachaux et al., 1999). The phase-locking value employed the amplitude of the first circular moment of the measured phase difference between two phases (Lachaux et al., 1999; Canolty et al., 2010). The phase-locking value ranges between 0 and 1; 0 signifying purely random rise and fall, whereas a value of 1 signifies that one signal perfectly follows the other. To distinguish between noise-related fluctuations of the phase-locking values we compared the observed data with shuffled data (Tsanov et al., 2014).

### Virus construction and optical activation

We used a Cre-inducible ChR2 viral construct designed for optogenetic purposes (Tsai et al., 2009; Witten et al., 2011). pAAV-Ef1a-DIO-hChR2(E123T/T159C)-EYFP-WPRE-pA was serotyped with AAV5 coat proteins and packaged by Vector Core at the University of North Carolina. Viral titers ranged from 1.5 to 8 × 10^12^ particles per mL (Witten et al., 2011). Control viral vector bearing only the YFP reporter confirmed no effect of laser light on the recorded septal neurons (Witten et al., 2011). The virus injection was applied in the medial septum (+0.5 AP, 1.1 ML, angle 10° medially), with volume of 2 μl injected on two levels: 1 μl at 5.0 and 1 μl at 6.0 mm dorsoventral to dura. Simultaneous optical stimulation and extracellular recording were performed in freely-behaving rats. The optical fiber (200 μm core diameter, Thorlabs, Inc.) was inserted inside the microdrive cannula (Axona, Ltd.) of the recording tetrodes, with the tip of the tetrodes projecting beyond the fiber by 500 μm, and the optical fiber was coupled to a 473 nm laser (Thorlabs, Inc.). The light power was controlled to be 10–20 mW at the fiber tip. Square pulse with duration of 5–10 ms was delivered at frequency of 8–10 or 40–50 Hz.

### Theta-burst stimulation

Due to the optogenetic channelrhodopsin kinetics, requiring pulse duration of >5 ms for efficient activation and recovery period of ~10 ms (Nagel et al., 2003; Boyden et al., 2005; Yizhar et al., 2011) we used electrode-mediated stimulation. Theta-burst stimulation (TBS) was delivered through electrodes (SNEX-300, Kopf Instruments) implanted together with the tetrodes in medial septum. TBS protocol was generated by a constant current bipolar stimulus isolator (A365D, World Precision Instruments, Inc.), which was controlled through TTL input from the recording system. TBS consisted of four bursts, with each burst containing 3 pulses at 10 ms (100 Hz), with an inter-train interval of 125 ms (8 Hz). The stimulus isolator was synchronized with the video-tracking and with the recoding system (Axona Ltd). The electric current stimulation allowed application of pulses with very short pulse duration (0.2 ms) for fast frequency protocols (Tsao et al., 2013). TBS intensities were in the range of 50–200 μA (Dragoi et al., 2003; Tsao et al., 2013) and were fine-tuned individually with respect to the amplitude of the test-pulse stimulus artifact.

### Spatially-tuned TBS protocol

The rectangular-shaped linear track was pixelated and customized scripts were developed to trigger TBS of amplitude-controlled pulses to medial septum efferents every time the rat entered the place field of chosen place cell. The stimulation and the subsequent off-line analyses investigated separately the place field properties clockwise and counter-clockwise directions for the bidirectional place cells. To reduce the distortion effect of very small and very large place fields on the intra-field analysis we have excluded place cells with directional place filed diameter >30 and <10 cm. The environment was divided and each pixel was a square or rectangle with 1.5 cm width. After the baseline session the coordinates of a chosen place field were identified and customized scripts were developed to trigger pulses when the animal enters these coordinates. During the TBS session the recording system identified on-line the change of the spatial positioning of the animal and the stimulus isolator triggered TBS when the animal crossed the identified pixels.

### Histology

At the end of the study, brains were removed for histological verification of electrode localization. Rats were deeply anesthetized with sodium pentobarbital (390 mg/kg) and perfused transcardially with ice-cold 0.9% saline followed by 4% paraformaldehyde (see Materials and Methods). Brains were removed, post-fixed in paraformaldehyde for up to 24 h and cryoprotected in 25% sucrose for >48 h. Brains were sectioned coronally at 40μm on a freezing microtome. Primary antibody incubations were performed overnight at 4°C in PBS with BSA and Triton X-100 (each 0.2%). The concentration for primary antibodies was anti-ChAT 1:200 (Millipore). Sections were then washed and incubated in PBS for 10 min and secondary antibodies were added (1:200) conjugated to Alexa Fluor 594 dye (Invitrogen) for 2 h at room temperature. For visualization, the sections were mounted onto microscope slides in phosphate-buffered water and cover-slipped with Vectashield mounting medium (Fisher Scientific). The YFP fluorescence was evaluated within a selected region that was placed below the fiber tip in area of 1.5 × 1.5 mm. Fluorescence was quantified based on the average pixel intensity within the selected region (Witten et al., 2011). The stained sections were examined with Olympus BX51 fluorescence microscope at 594 nm for Alexa Fluor secondary antibody and 488 nm for ChR2-YFP. ChAT-positive neurons were identified based on expression of red fluorescence, whereas ChR2-positive neurons were identified by expression of green fluorescence. Co-localization of Alexa Fluor 594 and YFP was determined manually using ImageJ software (Image Processing and Analysis in Java).

### Statistical analyses

All data were analyzed using Prism software (GraphPad Software, Inc., La Jolla, CA). Statistical significance was estimated by using two-tailed *t*-test, Wilcoxon signed rank rest for paired data, Mann–Whitney test for unpaired data and two-way analysis of variance (ANOVA) paired with *post-hoc* Newman–Keuls test. The probability level interpreted as significant was *p* < 0.05. All data points are plotted ± sem.

## Results

### Septal network response to optogenetic activation of cholinergic neurons

**Figure 1:**
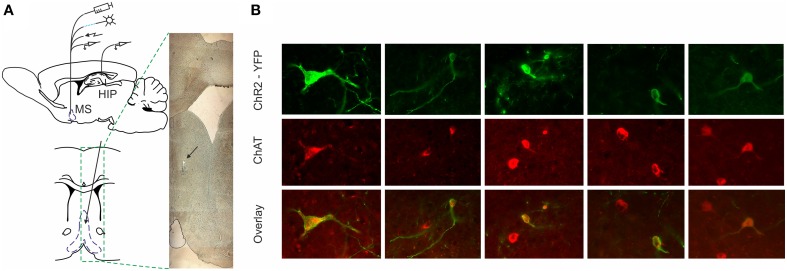
**ChR2-YFP expression in the medial septum of ChAT::Cre rats. (A)** Atlas schematic of our experimental setup investigating the functional relation of septal activity to the hippocampal formation (MS, medial septum; HIP, hippocampus). The medial septum of ChAT::Cre rats was injected with cre-inducible ChR2-EYFP. Chronically-implanted headstage with optic fiber and microdrive allowed parallel application of blue laser light and measurement of single unit activity in medial septum. Additionally, the implantation of bipolar concentric electrode allowed electric stimulation. Concurrently, recording tetrodes were implanted in hippocampal area CA1 to measure neuronal and local field activity. Coronal atlas schematic (below) and histological section (right) show where optic fiber and eight tetrodes were implanted and subsequently lowered in medial septum. The black arrow indicates the location of tetrodes tip. **(B)** Colocalization of ChAT staining and ChR2-YFP expression in the medial septum. High-magnification views of ChR2-YFP expression in ChAT-positive septal cell bodies after injection of cre-dependent virus in the medial septum of ChAT::Cre rats.

**Figure 2:**
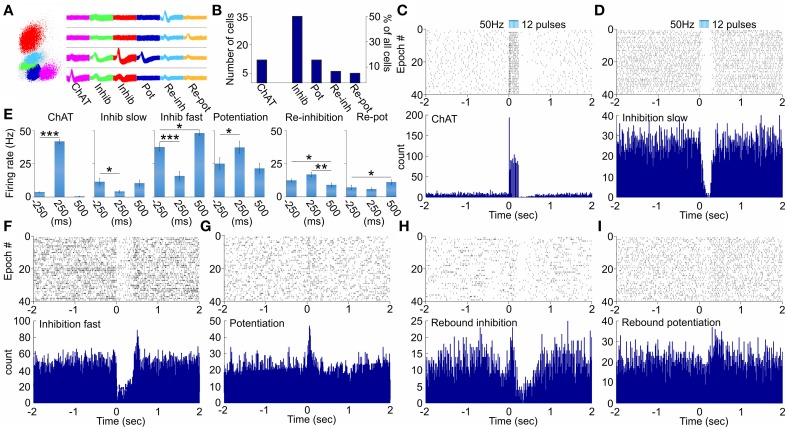
**Physiology of multiunit optical responses in medial septum of ChAT::Cre rats. (A)** Sample scatterplot, showing all signals recorded on a given tetrode. Right: sample waveforms of septal units, corresponding to neurons from the ChAT, inhibition (slow-spiking), inhibition (fast-spiking), potentiation, re-inhibition and re-potentiation groups. **(B)** Sample size of the main groups of neurons, which responded to optogenetic activation of septal cholinergic neurons. **(C)** Raster plot from 40 repetitions (above) and spike count of 120 repetitions (below) of optically evoked time-locked responses of ChAT cell. Time 0 indicates the delivery of the first train of the stimulation protocol (50 Hz, 5 ms pulse duration, 12 pulses, 473 nm). **(D)** Raster plot from 40 repetitions (above) and spike count of 120 repetitions (below) of slow-spiking unit from the inhibition group. **(E)** Comparison of the firing rate (spikes/s) 250 ms before, 250 ms after and 500 ms after the stimulation protocol for ChAT, slow-spiking inhibition, fast-spiking inhibition, potentiated, re-inhibition and re-potentiation neurons. Error bars represent ± sem, Wilcoxon signed-rank test; ^*^P < 0.05, ^**^P < 0.01, ^***^P < 0.001. Multiunit activity in response to optical activation of septal cholinergic neurons: sample raster (above) and spike count (below) plots recordings of fast-spiking inhibition **(F)**, potentiation **(G)**, re-inhibition **(H)**, and re-potentiation **(I)** units.

To distinguish the selective effect of septal cholinergic neurons on septo-hippocampal circuitry (Figure 1A) we used choline acetyltransferase (ChAT)::Cre rat line (Witten et al., 2011). Injection of a Cre-dependent adeno-associated virus in Chat::Cre rat lines resulted in highly specific expression of light-activated channelrhodopsin-2 tagged with a fluorescent protein (ChR2-YFP) in cholinergic neurons (Figure 1B, Supplementary Figure 1G). 90 ± 5% of neurons that expressed YFP also expressed ChAT, while 45 ± 5% of neurons that expressed ChAT also expressed YFP (*n* = 7 rats, *n* = 85 cells). We applied laser stimulation of septal cholinergic neurons in the animals implanted with optic fiber and recording tetrodes and evaluated the local network effect in medial septum. Trains of square pulses of blue light were applied continuously every 6 s. From 215 cells recorded in medial septum (*n* = 7 rats) we identified 70 cells that changed their firing frequency as a result of the stimulation (Figures 2A,B). Twelve cells (17% of the affected cells) responded with immediate robust increase of the firing frequency. The responsive neurons reliably followed trains of pulses and such light-entraining defines this group as putative ChAT neurons (Figure 2C, Supplementary Figure 1A). The relatively low number of recorded ChAT neurons reflects their population sparsity in medial septum (Witten et al., 2011). Thirty five cells (50%) responded with robust inhibition of the firing frequency (Figures 2D,E, Supplementary Figures 1C,E); the fast-spiking neurons from this group responded with instant firing suppression, followed by rebound post-stimulation facilitation (Figures 2F,E). Twelve cells (17%) responded with potentiation of the firing frequency that was following the stimulation onset with 4–14 ms (Figure 2G, Supplementary Figure 1B). Six cells (9%) responded with brief initial potentiation, followed by rebound inhibition (Figure 2H, Supplementary Figure 1D). Finally, five cells (7%) responded with rebound potentiation that was initiated after the stimulation protocol (Figure 2I, Supplementary Figure 1F). The last four groups, which comprise 83% (*n* = 58) of the affected cells, are defined as non-cholinergic neurons. The ChAT neurons group is characterized with tonic, regular slow-frequency firing 3.7 ± 1.4 Hz (Figure 2E). Based on their bursting firing patterns and high discharge rates >24 Hz (Bassant et al., 2005), we suggest that the non-cholinergic neurons from the potentiation and the fast-spiking inhibition groups are GABA-ergic cells. These data illustrate that selective cholinergic activation of septal cells triggers a wide intra-septal network effect expressed as differential responses from the non-cholinergic neurons. Thus, the hippocampal response to optogenetic ChAT-stimulation involves the indirect activation of large number of non-cholinergic septo-hippocampal projections.

### Stimulation frequency effect on the neuronal responsiveness in medial septum

Our next goal was to find the most efficient stimulation protocol that exerts the most powerful effect on septal neurons. First, we have established the light output power of 18 mW at the optic fiber tip as evoking maximal excitation response compared to lower intensities (Supplementary Figure 2A). We also established that the most optimal pulse duration is in the range of 5–10 ms (Supplementary Figure 2B). The next parameter of investigation was the stimulation frequency. We compared low (8–10 Hz) vs. high (40–50 Hz) stimulation frequency (Supplementary Figures 2C–E). The spiking of ChAT neurons was triggered consistently by both low and high frequency stimulation protocols. However, high frequency stimulation evokes greater response in the first 20 ms of the optogenetic stimulation (Supplementary Figure 2F), which include the first two pulses of 50 Hz protocol. The maximal spiking increase as percent of baseline was 2849.7 ± 508% in response to 50 Hz, compared to 1809.5 ± 169% during 10 Hz (*n* = 120 trials, *p* < 0.01, *t*-test, Supplementary Figure 2C). The non-ChAT neurons from the potentiation group (Supplementary Figure 2G) also expressed the higher degree of augmentation as a result of 50 Hz (181.8 ± 13.2%) compared to 10 Hz stimulation protocol (149.1 ± 8.9%; *n* = 120 trials, *p* < 0.05, *t*-test, Supplementary Figure 2D). Similarly, the neurons from the inhibitory group followed the tendency of more expressed spike suppression after 50 Hz protocol (18.8 ± 6.5%; Supplementary Figure 2H), compared to 10 Hz (36.9 ± 9.7%; Supplementary Figure 2E). While, the group with rebound potentiation (Supplementary Figure 2I) showed significantly higher response after high-frequency stimulation (168.5 ± 8.8% for 50 Hz and 147.6 ± 4.7% for 10 Hz; *n* = 120 trials, *p* < 0.05, *t*-test; Supplementary Figure 2D), the group with rebound inhibition did not show preference for the stimulation frequency (43.5 ± 7.6% for 50 Hz and 45.7 ± 9.7% for 10 Hz; Supplementary Figure 2E). These data illustrate the stimulation frequency plays a role in the ChAT-mediated network activation of septal neurons where higher frequency results in more potent network effect.

### Behavioral state dependence of the septal network responses

**Figure 3:**
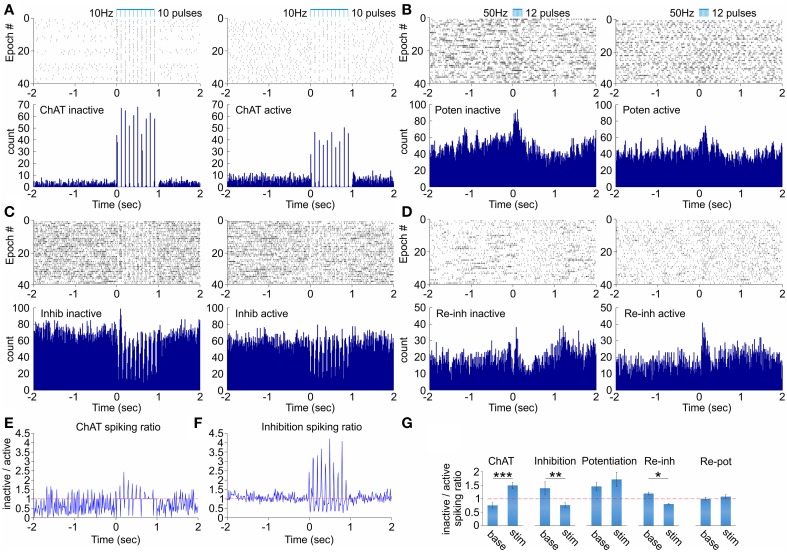
**Dependence of the septal network response on the behavioral state. (A)** Raster plot and spike count of representative ChAT cell during inactive (left) and active (right) behavioral state after 10 Hz stimulation protocol. **(B)** Raster plot and spike count of representative potentiation cell during inactive (left) and active (right) behavioral state after 50 Hz stimulation protocol. **(C)** Raster plot and spike count of representative inhibition cell during inactive (left) and active (right) behavioral state after 10 Hz stimulation protocol. **(D)** Raster plot and spike count of representative re-inhibition cell during inactive (left) and active (right) behavioral state after 50 Hz stimulation protocol. **(E)** Ratio of the spiking firing rate for inactive over active behavioral state for the ChAT unit shown in **(A)**. **(F)** Ratio of the spiking firing rate for inactive over active behavioral state for the inhibition unit shown in **(C)**. **(G)** Ratios of the spiking firing rate for inactive over active behavioral state for baseline background activity (left) and stimulation-induced spiking (right) for ChAT, inhibition, potentiation, re-inhibition, and re-potentiation groups. Error bars represent ± sem, paired *t*-test; ^*^P < 0.05, ^**^P < 0.01, ^***^P < 0.001. The horizontal red dotted line indicates the ratio level of 1.

The activity of cholinergic neurons in medial septum depends on the behavioral state of the animal (Marrosu et al., 1995; Jones, 2005). Thus, we explored next if the behavioral state exerts an effect on the septal network response to optogenetic ChAT stimulation. We compared the stimulation effect during active sessions (with average velocity of > 10 cm/s) vs. inactive or immobile sessions (with average velocity of < 2 cm/s). The neurons from the ChAT group expressed higher degree of activation during the inactive behavioral state (41.9 ± 1.4 Hz; Figure 3A, left) compared to the optogenetic response during pellet-chasing task (28.3 ± 1.3 Hz; Figure 3A, right). Concurrently, the background spiking activity of the ChAT neurons is lower during the inactive (3.4 ± 0.3 Hz), compared to active behavioral state (4.7 ± 0.3 Hz; Figures 3E,G). The fast-spiking neurons from the inhibition group showed more expressed suppression after stimulation during the inactive (15.8 ± 3.4 Hz, Figure 3C left) compared to the active states (19.7 ± 3.6 Hz; Figure 3C right, Figures 3F,G). The spiking frequency of the potentiation group of non-ChAT neurons underwent higher augmentation as a result of the stimulation protocol during inactive (37.3 ± 4.7 Hz, Figure 3B left), compared to the active behavioral state (21.8 ± 3.9 Hz, Figure 3B right). However, the baseline spiking from the inactive (25.11 ± 4.5 Hz) behavioral state also decreased during the active state (17.31 ± 3.8 Hz), thus, following similar ratio increase as the stimulation-evoked rate (Figure 3G). The most apparent dependence of the behavioral state was expressed by the non-ChAT neurons from the group responding with initial potentiation, followed by rebound inhibition (Figure 3D). Their background firing rate decreased during the active (10.5 ± 1.2 Hz) compared to inactive state (12.4 ± 1.1 Hz). However, the stimulation-induced potentiation was higher during active (21.0 ± 1.5 Hz) vs. inactive state (16.8 ± 1.7 Hz; Figure 3G). Furthermore, their spiking frequency showed preference for rebound decrease only during the inactive state (Figure 3D, left). The non-Chat neurons that respond with rebound potentiation did not express significant difference between the behavioral states (9.9 ± 2.7 Hz for inactive; 9.2 ± 2.8 Hz for inactive Figure 3G). Our data show that the behavioral state influences the efficiency of optogenetic-induced activation of the ChAT neurons. These findings support the idea that cholinergic neurons are already highly-activated during active exploration and, thus, additional activation of septal ChAT neurons has reduced hippocampal effect (Vandecasteele et al., 2014).

### Medial septum stimulation results in differential effect of hippocampal interneurons

**Figure 4:**
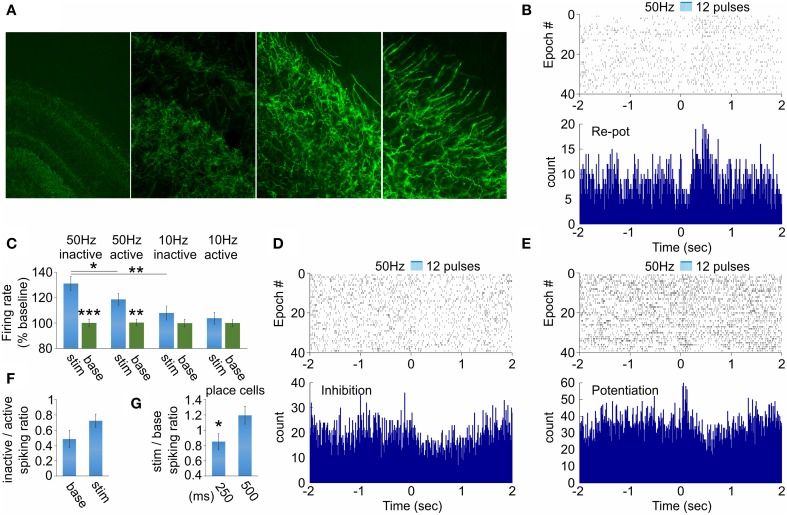
**Hippocampal neuronal responses after septal ChAT stimulation. (A)** High-magnification views of septal axons expressing ChR2-YFP in hippocampal CA1 area after injection of cre-dependent virus in the medial septum of ChAT::Cre rats. **(B)** Raster plot from and spike count of optically evoked responses from hippocampal re-potentiation unit. Time 0 indicates the delivery of the first train of the stimulation protocol (50 Hz, 5 ms pulse duration, 12 pulses, 473 nm). **(C)** Average values of the stimulation-evoked firing rate increase (as percent of the baseline firing rate) for the potentiation and re-potentiation hippocampal units for stimulation frequency vs. behavioral state: 50 Hz inactive, 50 Hz active, 10 Hz inactive and 10 Hz active, respectively. Error bars represent ± sem, Wilcoxon signed-rank test; ^*^P < 0.05, ^**^P < 0.01, ^***^P < 0.001. Raster plots from and spike count of optically evoked responses from hippocampal inhibition **(D)** and potentiation **(E)** units. **(F)** Ratio of the firing rate for inactive over active behavioral state for baseline background activity (left) and stimulation-induced spiking (right) for hippocampal interneurons. **(G)** Ratio of the firing rate for stimulation over baseline for 250 ms (left) and 500 ms (right) after septal stimulation for hippocampal place cells. Error bars represent ± sem, paired *t*-test; ^*^P < 0.05.

Our next goal was to examine how hippocampal function responds to septal activation. We evaluated the effect of behavioral state and stimulation frequency variables on (1) the spiking of hippocampal neurons and (2) theta band (5–12 Hz) of hippocampal local field oscillations. The optic fiber was located in medial septum, while the recording tetrodes were located in dorsal hippocampal CA1 area (see Figure 1A). We have chosen the stimulation locus to be in the medial septum but not in hippocampus to avoid the photoelectric effect on the hippocampal local field potential (Cardin et al., 2010). In the hippocampal recording site we identified cholinergic septal efferents expressing ChR2-YFP (Figure 4A). Therefore, the evoked hippocampal response involves direct activation of ChAT projections together with indirect activation of non-ChAT septal neurons. We first analyzed how septal cholinergic activation alters the spiking of hippocampal interneurons. From 68 fast-spiking cells recorded in the dorsal hippocampus of three rats we observed stimulation-induced change of the spiking of 17 neurons. Of these 17 neurons, 11 neurons responded with initial inhibition and rebound potentiation (Figure 4B), four neurons responded with inhibition (Figure 4D) and two responded with potentiation followed by rebound inhibition (Figure 4E). We compared low (10 Hz) vs. high (50 Hz) stimulation and active vs. inactive behavioral state. This approach results in four configurations: (1) low frequency × active, (2) low frequency × inactive, (3) high frequency × active, (4) high frequency × inactive. The spiking of hippocampal neurons was significantly increased only by 50 Hz septal stimulation (Figure 4C, Supplementary Figure 3A). Concurrently, inactive behavioral state showed higher spiking increase (131 ± 5.6%, *n* = 120 trials, *p* < 0.01, Wilcoxon signed-rank test, Figure 4C, Supplementary Figures 3A,B) compared to active behavioral state (118 ± 4.6%, *n* = 120 trials, *p* < 0.05, Wilcoxon signed-rank test, Figure 4C, Supplementary Figures 3C,D). The spiking of hippocampal interneurons was increased during the active state and this is represented by the low levels of inactive/active spiking ratio (0.48 ± 0.11). The stimulation protocol induced augmentation of inactive/active spiking ratio (0.72 ± 0.09), although the values remained below 1 (Figure 4F). We also examined the spiking of the complex-spike cells expressing spatial properties during the active state (*n* = 6 cells). The firing frequency of the place cells was significantly affected only for the inactive state, where the stimulation/baseline spiking ratio decreased during the 50 Hz stimulation, compared to the ratio after the stimulation protocol (*n* = 120 trials, *p* < 0.05, *t*-test, Figure 4G). These data reveal a differential response of the hippocampal neurons to the septal stimulation, and this response is frequency-dependent. Additionally, the selective septal cholinergic activation is insufficient to trigger robust effect on hippocampal neurons during the active behavioral state.

### Cholinergic septal activation evokes frequency-dependent and behavioral state-dependent effect of hippocampal oscillations

**Figure 5:**
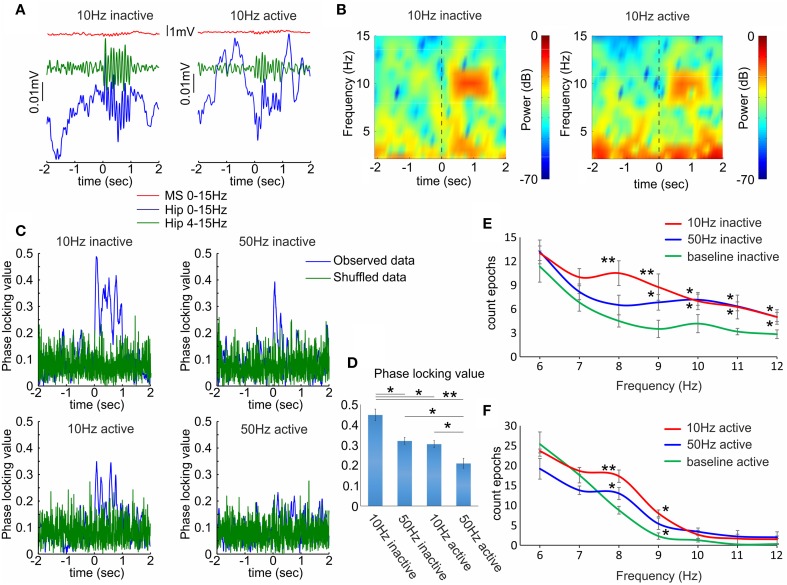
**Hippocampal local field responses after septal ChAT stimulation. (A)** Sample event related potentials (ERP) recorded in dorsal CA1 after 10 Hz septal optogenetic stimulation during inactive (left) and active (right) behavioral state. Upper red traces show ERP from medial septum, middle green traces represent ERP from hippocampus band-pass filtered (4–15 Hz) and lower blue traces represent the same hippocampal ERP after low-pass filtered (0–15 Hz). Time 0 indicates the delivery of the first train of 10 Hz stimulation protocol to medial septum. **(B)** Color-coded power spectrograms of hippocampal low-frequency oscillations after 10 Hz septal stimulation protocol during inactive (left) and active (right) behavioral state. Vertical dotted black line indicates time 0. **(C)** Representative samples of phase-locking value for 10 Hz inactive (top, left), 50 Hz inactive (top, right), 10 Hz active (below, left), and 50 Hz active (below, right) state. Blue traces show the observed data, while the green values represent shuffled data. **(D)** Average values of the phase-locking value for the same groups. Error bars represent ± sem, two-tailed *t*-test; ^*^P < 0.05, ^**^P < 0.01. **(E)** Frequency histogram of band-passed local field potential for 10 Hz (red), 50 Hz (blue) and baseline epoch counts (green) during inactive behavioral states. **(F)** Frequency histogram of band-passed local field potential for 10 Hz, 50 Hz and baseline epoch counts during active behavioral states. Error bars represent ± sem, Newman–Keuls test, ^*^P < 0.05.

Similarly to the septal neuronal response the behavioral state modified the effect of septal optogenetic stimulation on the hippocampal theta oscillations. Theta increase was significantly higher during inactive (Figures 5A,B left panels), compared to active recording sessions (Figures 5A,B right panels). The most potent effect on hippocampal theta was observed after low-frequency stimulation protocol (8–10 Hz). For better trace visualization we averaged multiple trials to generate event related potentials and remove uncorrelated activity (Figure 5A). We also evaluated the effect of optogenetic stimulation on the synchronization of the local field potential across the stimulation trials (*n* = 120) in four rats. We used the phase-locking value (see Materials and Methods), which measures the degree of local field synchrony between all stimulation epochs in the theta frequency range. We analyzed the values for the first 100 ms of the stimulation protocols. The phase-locking values were higher for 10 Hz (0.44 ± 0.02; Figure 5C top, left) compared to 50 Hz (0.32 ± 0.01; Figure 5C top, right) during inactive states (*n* = 120 trials, *p* < 0.05, *t*-test, Figure 5D). Similarly, the value was higher for 10 Hz (0.30 ± 0.04; Figure 5C below, left) compared to 50 Hz (0.21 ± 0.02; Figure 5C below, right) during the active states (*n* = 120 trials, *p* < 0.05, *t*-test, Figure 5D). The increase of theta amplitude was significantly higher after 10 Hz stimulation, compared to 50 Hz stimulation (*n* = 120 trials, *p* < 0.05, Newman–Keuls test) for both inactive (Figure 5E) and active states (Figure 5F). We did not analyze the correlation values between the laser-induced septal and hippocampal theta oscillations because of the interference of the photoelectric effect in the local field potentials (Supplementary Figure 4). These data confirm the relevance of the behavioral state for the cholinergic septo-hippocampal processing. Unlike the neuronal response in medial septum and hippocampus to high frequency stimulation, the hippocampal theta synchronization was preferentially altered after low frequency protocol. Our findings suggest that the most efficient synchronization of hippocampal theta rhythms requires septal stimulation pattern with inter-stimulation intervals of ≤10 Hz.

### Non-selective theta-burst stimulation of medial septum evokes concurrent neuronal and theta response during active exploration

**Figure 6:**
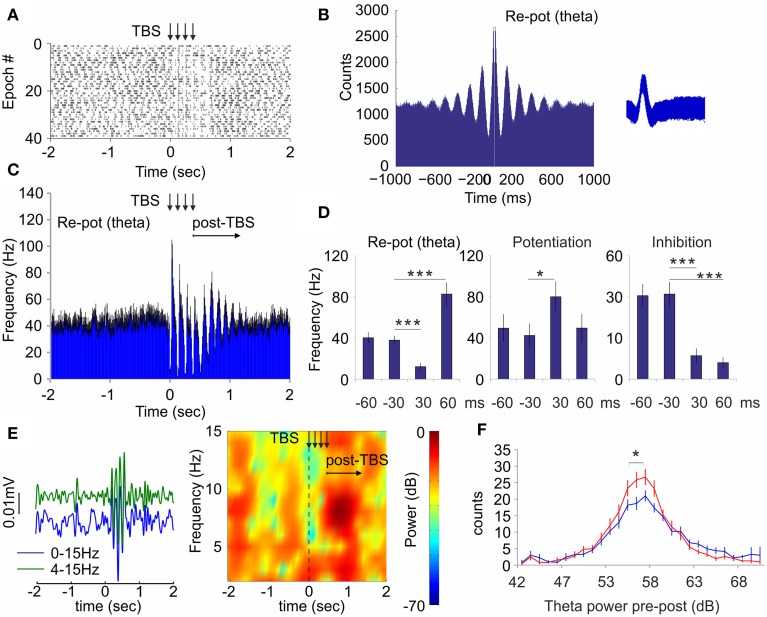
**Theta-burst stimulation of medial septum resets the spiking of hippocampal interneurons and theta oscillations. (A)** Raster plot and spike count of representative re-potentiation hippocampal cell after electric theta-burst stimulation protocol (TBS, four bursts with inter-train interval of 100 Hz and inter-train interval of 8 Hz) to medial septum. Time 0 indicates the delivery of the first train. The vertical arrows indicate the delivery time of four trains. **(B)** Sample of 1000 ms autocorrelogram and spike waveform (right) of hippocampal theta unit. **(C)** Averaged frequency histogram for all theta cells. The vertical arrows indicate the delivery time of four trains. The horizontal arrow indicates the post-stimulation period. Black error bars represent ± sem. **(D)** Averaged firing frequency from −60 ms before to 60 ms after the first train for re-potentiated (theta), potentiated, and inhibited units, respectively. Error bars represent ± sem, Mann–Whitney test; ^*^P < 0.05, ^***^P < 0.001. **(E)** Left: Sample band-pass filtered and low-pass filtered ERP recorded in dorsal CA1 after septal TBS. Right: Sample color-coded power spectrogram of the same recording. **(F)** Power histogram of hippocampal theta power before (blue) and after (red) TBS. Error bars represent ± sem, Two-Way ANOVA, ^*^P < 0.05.

While the local field response is favored by low frequency, the neuronal response in hippocampus is more effectively triggered by high frequency (see Figure 4). To combine both frequency modes into one stimulation protocol we next stimulated septal afferents with electric current via bipolar concentric electrode. This approach allowed us to apply theta-burst stimulation protocol with short pulse duration (0.2 ms), high intra-burst frequency (100 Hz) in parallel with low inter-burst frequency (8 Hz, see Materials and Methods). Theta-burst stimulation (TBS) protocol mimics most closely the spiking pattern of septal theta-bursting cells (Fking et al., 1998; Tsanov et al., 2014). Theta-burst spiking is a feature of GABA-ergic cells in the medial septum (Bassant et al., 2005). The stimulation was delivered to the animals (*n* = 4 rats) in active behavioral state during pellet-chasing task. Similarly to the optogenetic septal stimulation we observed three types of responses from the hippocampal interneurons: (1) group of neurons that responded with initial inhibition, followed by subsequent potentiation (*n* = 11 cells, Figure 6A); (2) group of neurons that responded with potentiation (*n* = 6 cells, Supplementary Figures 5A,B); and (3) group of inhibited neurons (*n* = 5 cells, Supplementary Figures 5C,D). The majority cells from the re-potentiation group are spiking with theta-bursting patterns (*n* = 9, theta cells; Figure 6B). Application of TBS entrained the spiking of the theta cells, leading to synchronization of their spiking that continued for 600–900 ms after TBS (Figure 6C). The high-frequency stimulation pattern induced intra-burst suppression of the theta cells' spiking (12.5 ± 3.5 Hz) and inter-burst augmentation (82.6 ± 11.4 Hz), compared to baseline levels (40.6 ± 5.2 Hz; Figure 6D, Supplementary Figures 5E,F). The potentiation group of neurons showed significant response only to the intra-burst period of TBS (80.1 ± 14.7 Hz) compared to the pre-stimulation rate (42.4 ± 11.4 Hz, Mann*–*Whitney, *n* = 120 trials, *p* < 0.05; Figure 6D, Supplementary Figures 5G,H). The temporal overlap of the spiking changes for re-potentiation (theta) and potentiation groups (Supplementary Figures 5E,G) suggests a functional connection between them. Finally, the inhibition group of cells undergo suppression throughout the stimulation protocol (Figure 6D, Supplementary Figure 5I), which was more expressed during the intra-burst periods (Supplementary Figure 5K). Concurrently, the electrical stimulation of medial septum also increased hippocampal theta power (Figure 6E). TBS resulted in a parallel increase in hippocampal theta power (Figure 6F, Two-Way ANOVA, *F*_(1, 28)_ = 1.730, *n* = 120 trials *p* < 0.05), which occurred at the end of TBS protocol and shortly after TBS (Figure 6E), following similar timing with the post-TBS synchronization of re-potentiation group of cells (Figure 6C). These findings demonstrate that electrical TBS protocol efficiently entrains the hippocampal neuronal activity during pellet-chasing task with the same differential neuronal responses as septal optogenetic ChAT-stimulation but with better temporal resolution. Our data show that TBS evokes potent hippocampal response, where theta oscillations are closely-linked to the intrinsic synchronization of the hippocampal theta cells.

### Theta-burst stimulation exerts timing-dependent effect on the hippocampal place fields

**Figure 7:**
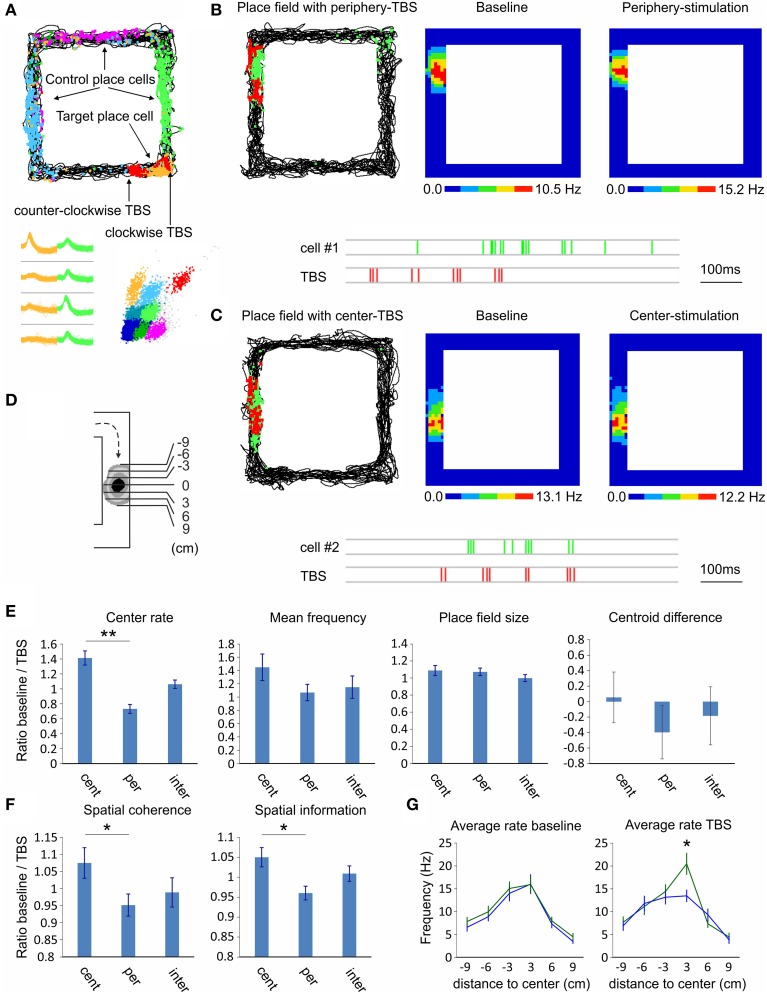
**Septal TBS effect on hippocampal place cells. (A)** Sample place field maps of CA1 pyramidal neurons after exploration of rectangular-shaped linear track. The non-stimulated place cells (blue, purple, and green) are denoted as controls, while the target place cell (yellow) is stimulated with septal TBS (red). TBS pulses are positioned on each side of the field depending on the direction (clockwise and counter-clockwise) of the animal in the track. Bottom: spike waveforms and spike clusters of the same place cells. **(B)** Sample of periphery-stimulated place cell (cell #1); Left: map of animal trajectory with spikes (green) and TBS pulses (red). Firing rate maps during baseline (middle) and TBS session (right). Below: sample recording of the place cell spikes (green) and the preceding TBS pulses (red). **(C)** Sample of center-stimulated place cell (cell #2); map of animal trajectory with spikes (green) and TBS pulses (red). Note that the TBS pulses targeted the center of the place field in the course of clockwise direction. Firing rate maps during baseline (middle) and TBS session (right). Below: sample recording of the place cell spikes (green) and TBS pulses (red). **(D)** Scheme of place field binning for clockwise direction. **(E)** Comparison of the place field properties between TBS applied in the center of the place field (−3 to 0), in the periphery of the place field (−9 to −6), or intermediately (−6 to −3). Center rate, mean firing frequency, place field size and centroid difference **(E)**, and spatial coherence and spatial information content **(F)** are represented as ratios of the measured values from baseline session over the values of the TBS session. **(G)** Average intra-field firing rate per 3 cm bins during baseline (left) and TBS (right) sessions, for periphery-stimulated (green) and center-stimulated (blue) place fields. Error bars represent ± sem, Newman–Keuls test; ^*^P < 0.05, ^**^ *P* < 0.01.

Our final goal was to investigate the effect of septal activation on the hippocampal pyramidal neurons and their place field properties. For this purpose we conducted two subsequent recordings in rectangular-shaped linear track (Figure 7A); the first recording was baseline session and the subsequent one was TBS session. During the TBS session we applied the stimulation protocol every time the animal approached the place field of a chosen cell (see Materials and Methods). TBS evoked no change in the behavior or in the velocity of the animals (15.1 ± 3.7 cm/s for baseline session and 14.8 ± 3.2 cm/s for TBS session). We compared effect of TBS protocol applied at the periphery of the place fields, *n* = 35 cells (9 to 6 cm prior the place field center, Figure 7B; −9 to −6 cm in Figure 7D), to the effect of the protocol when applied at the center of the place fields, *n* = 23 cells (3 to 0 cm prior the place field center, Figure 7C; −3 to 0 cm in Figure 7D). In the cases where TBS was applied intermediately (6 to 3 cm prior the place field center; −6 to −3 cm in Figure 7D) were defined as a separate group (*n* = 20 cells). We also analyzed the firing properties of the targeted unit with the firing properties of the units with place fields in a distal location (controls). We investigated the mean and center firing rate, the spatial coherence, the spatial specificity (or spatial information content), field size and centroid difference between clockwise and counter-clockwise directions. We represented the CA1 place field properties as ratios of the measured values from baseline session over the values of the TBS session. Our data show significant difference of the place field center rate between the periphery-stimulated fields and the center-stimulated fields (Wilcoxon signed-rank test, *n* = 4 rats, *p* < 0.01; Figure 7E; Supplementary Figure 6). While the firing rate of periphery-stimulated field increases the same parameter decreases for the center-stimulated fields. The intermediate-stimulated group shows baseline/TBS ratio close to 1. Mean firing frequency of the stimulated place cells shows non-significant tendency, while the place field size and centroid difference show no significant change between both groups (Figure 7E). The spatial coherence and the spatial information increase for the periphery-stimulated fields, while these parameters decrease for the center-stimulated fields (Wilcoxon signed-rank test, *n* = 4 rats, *p* < 0.05; Figure 7F). The control place cells show ratios close to 1 for all parameters with no significant change between sessions. One-dimensional binning analysis of the stimulated place fields reveals that the difference between the periphery-stimulated and center-stimulated fields is in the bin of 0 to 3 cm post place field center (Newman–Keuls, *n* = 4 rats, *p* < 0.05; Figure 7G). Considering the average velocity of our rats during the pellet-chasing task of 14.8 cm/s, this bin represents the end of TBS protocol and the onset of the post-TBS period for periphery-stimulated fields, while for center-stimulated fields it corresponds to the onset of TBS protocol. For the duration of TBS protocol (405 ms) the animals passed on average 5.9 cm. These data demonstrate that septal activity is able to control the spiking of hippocampal place cells and up-regulate or down-regulate the spatial representation, depending on the temporal proximity between septal activity and place field center.

## Discussion

We show here that optogenetic septal ChAT stimulation evokes a differential cholinergic and non-cholinergic neuronal response in medial septum of behaving transgenic Chat::Cre rats. This activation exerts frequency- and behavior-dependent effect on hippocampal formation. We provide evidence that the selective cholinergic activation is not sufficient to regulate the hippocampal function during active behavioral state. Non-selective septal theta-burst stimulation entrains the hippocampal neurons and regulates timing-dependently the place field spatial properties during active exploration. Our findings provide crucial evidence that medial septum is able to regulate hippocampal function on spatiotemporal scales much finer than previously known.

### Network effect of septal cholinergic stimulation

Here, we used recently-developed genetically-validated recombinase-driver rat line to target cholinergic septal cell types (Witten et al., 2011). This technique allowed us to combine ChAT-triggered perturbation of septo-hippocampal neural populations together with differing behavioral states with sufficient spatial sampling of the explored environment. The expression of light-activated channelrhodopsin-2 (ChR2-YFP) in ChAT neurons is reliable with 90% specificity in cholinergic neurons of the medial septum in Chat::Cre rats (Witten et al., 2011). The development of novel transgenic rat line is essential methodological advance for investigation of spatial navigation in rodents. However, Witten et al did not show optogenetic activation of the septal ChAT neurons in behaving rats. They reported *in vitro* current-clamp recording of light-evoked action potentials of ChAT cells and indirect effect *in vivo* expressed by inhibitory response (Witten et al., 2011). Here, we show for the first time the selective entrainment of ChAT neurons by blue light in behavioral context, revealing the practical value of this transgenic rat line. Furthermore, our data demonstrate that activation of septal cholinergic neurons evokes potent network effect in medial septum expressed in dissimilar response of the affected non-cholinergic neurons. The ChAT-triggered network effect is expressed predominantly by inhibition (35/50%). Smaller population of neurons responded with ChAT-evoked potentiation (12/17%). The reciprocal network effect is completed by rebound inhibition (6/9%) and rebound potentiation (5/7%). Only slow-firing neurons <4 Hz were entrained by the selective optogenetic protocol suggesting that the expression of the cre-promoted hChR2-YFP virus was restricted to cells with cholinergic profile (Simon et al., 2006). This was confirmed by the labeling of ChAT-positive cells in our histology. The fast-spiking potentiation units were not entrained by the optogenetic pulses, showing that the recorded signal was not result of non-specific viral expression. Furthermore, the delayed response of >4 ms for the potentiation cells suggests polysynaptic latency. There are limited data about intra-septal communication suggesting reciprocal connections between medial septal cholinergic and GABA-ergic neurons (Leranth et al., 1992; Manseau et al., 2005). Our findings provide evidence for intra-septal functional connectivity where at least 52% of the affected neurons (36 cells from the potentiation and the fast-spiking inhibition groups) share the firing patterns of GABA-ergic neurons (Bassant et al., 2005). The strong network effect of the optogenetic stimulation (83% of the affected cells, are defined as non-cholinergic neurons) suggests that the optogenetic ChAT activation evokes complex hippocampal response, which is triggered by cholinergic and non-cholinergic (inhibitory and probably glutamatergic) projections. This observation is confirmed by our finding that both selective optogenetic and non-selective electrical protocols lead to similar responses of hippocampal interneurons, including re-potentiation, potentiation and inhibition. Some of the non-cholinergic neurons affected by the optic activation of cholinergic neurons in the medial septum are very likely to be glutamatergic. Neurons, using vesicular glutamate transporter 2 (VGLUT2), are estimated to constitute 25% of the medial septum-diagonal band of Broca population (Colom et al., 2005). Fast-firing VGLUT2-positive neurons with spontaneous spiking up to 16 Hz *in vitro* also display rhythmic firing properties in theta range (Huh et al., 2010). The complexity of septo-hippocampal processing is complemented by the co-synthesis of glutamate in cholinergic and GABAergic neurons (Manns et al., 2001; Gritti et al., 2006).

### Behavior and stimulation frequency determine the efficiency of septal optogenetic effect

It is well-established that cholinergic transmission in the limbic system undergoes state-dependent variation. Acetylcholine release during active waking is increased by approximately 75% compared to quiet waking (Marrosu et al., 1995). To identify the behavioral effect on optogenetic activation we have applied the stimulation protocol during active (active waking) and inactive (quiet waking) behavioral states. We found that cholinergic activation in septo-hippocampal networks is activity-dependent with stronger response during the inactive behavioral states. The ratio of the stimulation-induced effect during the inactive state was augmented for majority of the recorded cells, including the ChAT group. The spontaneous firing frequency of the ChAT neurons in our recordings was 3.4 Hz during inactive state and 4.7 Hz during active behavioral state, consistent with previous data about the low-firing patterns of septal cholinergic neurons (Simon et al., 2006; Zhang et al., 2011). Altogether these results provide key evidence that the cholinergic neurotransmission has already reached high levels of muscarinic receptors activation during an active state and, therefore, the additional optogenetic stimulation of septal ChAT neurons had reduced impact. Such hypothesis was suggested in a recent study showing that, in waking, exploring mice the impact of optogenetic cholinergic stimulation on theta oscillations was less expressed compared to the same protocol in urethane-anaesthetized mouse (Vandecasteele et al., 2014). Our data also show that the optogenetic stimulation of medial septum did not exert a consistent effect on locomotion velocity and motor behavior. This is in agreement with previous observations (Bland et al., 2006; Vandecasteele et al., 2014). In addition, we explored which stimulation frequency is able to evoke the most efficient cholinergic response and network effect in medial septum. We established that both high and low frequencies evoked reliable response in septal neurons. However, the maximal firing rate of the ChAT neurons as well as the neurons from the potentiation and re-potentiation groups was higher for the 50 Hz compared to 10 Hz stimulation. Concurrently, the hippocampal neurons responded with significant change of their firing rates after 50 Hz but not after 10 Hz optogenetic septal stimulation. Medial septum, however, is not strictly related to switching behavioral states and theta states are characterized by increased release of acetylcholine that varies in a task-dependent manner on the time scale of seconds (Parikh et al., 2007; Zhang et al., 2010). Cholinergic septal cells activate interneurons under a fear condition, leading to inhibition of pyramidal distal dendrites at a very fast time scale (Lovett-Barron et al., 2014; Lovett-Barron and Losonczy, 2014). Thus, it is important to understand if cholinergic activation follows the fast GABA-ergic septo-hippocampal signaling during active behavior (Kaifosh et al., 2013).

### Septal stimulation effect on hippocampal single unit and local field activity

Our goal here was to investigate the effect of septal activation on hippocampus on a systems level; how the activation of septal network alters hippocampal neuronal, oscillatory and spatial properties. Considering the complexity of septo-hippocampal transmission—wide range of receptor classes and subtypes and their localization at both pre- and postsynaptic sites on both pyramidal cells and interneurons (Teles-Grilo Ruivo and Mellor, 2013), the exploration of the individual receptor-mediated contribution is more suitable for *in vitro* studies. Data from hippocampal intracellular recordings showed that cholinergic afferent stimulation elicits atropine-sensitive differential synaptic responses in CA1 interneurons (Widmer et al., 2006). The main responses were shown to include slow depolarization, slow hyperpolarization and biphasic membrane potential change in which an initial slow hyperpolarization subsequently transforms into a slow depolarization. Our findings demonstrated similar segregation of hippocampal neuronal responses to selective septal optogenetic as well as to the non-selective electric stimulation. Here, we have identified neurons responding with potentiation, inhibition and biphasic response, including initial inhibition and subsequent potentiation. Neuromodulatory cholinergic inputs from the medial septum regulate septo-hippocampal synchronization in response to behavioral states, by exercising control over the amplitude of theta rhythm in the limbic circuitry (Vinogradova, 1995; Vertes and Kocsis, 1997). We show here that selective ChAT septal stimulation affects hippocampal oscillations in theta range in a behavior-dependent manner. Similarly, the optogenetic stimulation-induced spiking of hippocampal neurons was behavior-dependent with significant change predominantly during the inactive sessions. Only fast non-selective septal stimulation resulted in resetting the bursting of hippocampal theta cells during active behavior, in parallel with increase of the phase-locking value that represents the degree of local field synchronization. Our data complement previous reports that electrical fornix stimulation may reset the hippocampal theta rhythm (Williams and Givens, 2003; Scarlett et al., 2004; Bland et al., 2006).

### Theta-burst stimulation of septo-hippocampal projections

We show here also frequency-dependent effect of septal stimulation on hippocampal theta rhythm. This finding provides a key evidence that the timing of inter-stimulation periods is essential parameter for the septo-hippocampal dynamics of theta generation. The local field synchronization expressed a preference for low-frequency stimulation protocol, whereas the hippocampal neuronal response showed significant increase after 50 Hz but not after 10 Hz optogenetic septal stimulation. Such dissimilar rate preferences suggest two components in the septo-hippocampal dialog. While slow-spiking septal ChAT neurons are linked to the amplitude of theta rhythm (Lee et al., 1994) by tonically depolarizing pyramidal cells and basket interneurons (Chapman and Lacaille, 1999), the fast-spiking septal GABA-ergic cells are linked to the frequency of theta rhythm by periodically hyperpolarizing hippocampal basket cells and rhythmically disinhibiting the pyramidal cells (Toth et al., 1997). Our findings suggest that the most optimal septal stimulation protocol should mimic the slow ChAT and the fast GABA-ergic firing patterns, thus, including both low- and high-frequency ranges. The ability to mimic *in vivo* patterns of septal theta-bursting neurons is critical for identifying and dissecting the physiological effects of medial septum on hippocampal function. Based on the discharge rates of the septal theta-bursting cells (Tsanov et al., 2014), we timed the inter-stimulation periods with the average duration of a theta cycle (8 Hz), together with high intra-burst stimulation frequency (100 Hz). Although optogenetic tools allow selective cholinergic stimulation, their frequency application currently is restricted due to the channelrhodopsin kinetics, which require pulse duration of >5 ms for efficient activation and recovery period of ~10 ms (Nagel et al., 2003; Boyden et al., 2005; Yizhar et al., 2011). While the selective optogenetic stimulation allowed us to identify the frequency- and behavioral effect of cholinergic component on the network responses in medial septum and hippocampus, the non-selective electric stimulation permitted the application of pulses with high frequency and precise temporal resolution during active exploration. Here, we used the two complementary approaches to examine the septal effect on hippocampal circuitry. We have shown that cholinergic stimulation during active behavioral state has insufficient effect on the hippocampal neuronal and local field potential, compared to the inactive state. TBS depolarized larger population of non-cholinergic septal projections, allowing us to entrain the spiking of hippocampal neurons during active exploration. TBS protocol successfully reset the bursting of hippocampal theta cells together with synchronization of local field potentials. This effect continued for 600–900 ms after TBS and this period was paralleled by increase of theta power.

### Theta-burst stimulation regulates the hippocampal spatial properties

Our methodological approach allowed us to stimulate hippocampal place cells depending on the spatial location of the animals. This allowed us to study the alteration in spike pattern of place cells as a result of septal stimulation in a particular pixel of the explored environment. Our data show that the effect of TBS on hippocampal place fields depends on the timing of the applied protocol. When TBS is applied 6–9 cm prior the center of the place field or 400–650 ms prior to the peak (periphery-stimulated fields), the center firing frequency increases. Importantly, the timing of increased place cell spiking coincides with intrinsically-entrained synchronization of hippocampal theta cells and concurrent theta power increase. The center-stimulated fields reflect spiking decrease that parallels the timing of extrinsically-induced entrainment of septo-hippocampal circuitry. This result suggests that stimulation of the medial septum activates the hippocampal neural circuitry involved in the generation of theta field activity in a non-physiological manner, dissociated from the normal behavioral correlates (Bland et al., 2006). We demonstrate that the spatial coherence and spatial information content increase for periphery-stimulated fields, while these parameters decrease for the center-stimulated fields. Spatial information and spatial coherence reflect the signal to noise relation of the place cells activity and are precise indicators for the encoding of hippocampal spatial representation (Poucet et al., 1991; Calton et al., 2003; Hok et al., 2013). Place field size and centroid location showed no significant change for both groups, demonstrating that that the septal stimulation is not able to remap the hippocampal place fields. Therefore, septo-hoppocampal signal processing do not alter the fundamental encoding properties of spatial representation. Hippocampal place fields that are formed internally as a result of network interactions during wheel running (Pastalkova et al., 2008) are abolished by the suppression of septal activity (Wang et al., 2015). Similarly the grid cells are not sustained during septal inactivation (Brandon et al., 2011; Koenig et al., 2011). However, this might not be the case with sensory-generated place fields which are formed in new environment during septal inactivation (Brandon et al., 2014). This line of research suggests that theta sequences are vital for the internally-generated firing fields and that theta sequences may mediate the formation of memory traces (Robbe and Buzsaki, 2009). Our results reveal several insights on the functions and mechanisms into the septo-hippocampal network regulation and provide a foundation for investigations into the circuit mechanisms of septal contributions to spatial navigation and memory.

## Conclusion

Our data show that the modulatory role of septal cholinergic control over the hippcampal place field representation is mediated by the rhythmic entrainment of hippocampal neurons and the ability of septo-hippocampal circuits to resonate in theta frequency. We used specific optogenetic stimulation of the septo-hippocampal cholinergic neurons in transgenic rats and non-specific theta-burst stimulation protocols to demonstrate that medial septum is a major player not only in the transition between behavioral states but also enables rapid regulation of the hippocampal spatial encoding.

### Conflict of interest statement

The authors declare that the research was conducted in the absence of any commercial or financial relationships that could be construed as a potential conflict of interest.

## References

[B1] Bassant M. H. Simon A. Poindessous-Jazat F. Csaba Z. Epelbaum J. Dournaud P. (2005). Medial septal GABAergic neurons express the somatostatin sst2A receptor: functional consequences on unit firing and hippocampal theta. J. Neurosci. 25, 2032–2041. 10.1523/JNEUROSCI.4619-04.2005 15728843 PMC6726075

[B2] Bland B. H. Bird J. Jackson J. Natsume K. (2006). Medial septal modulation of the ascending brainstem hippocampal synchronizing pathways in the freely moving rat. Hippocampus 16, 11–19. 10.1002/hipo.20136 16270325

[B3] Boyden E. S. Zhang F. Bamberg E. Nagel G. Deisseroth K. (2005). Millisecond-timescale, genetically targeted optical control of neural activity. Nat. Neurosci. 8, 1263–1268. 10.1038/nn1525 16116447

[B4] Brandon M. P. Bogaard A. R. Libby C. P. Connerney M. A. Gupta K. Hasselmo M. E. (2011). Reduction of theta rhythm dissociates grid cell spatial periodicity from directional tuning. Science 332, 595–599. 10.1126/science.1201652 21527714 PMC3252766

[B5] Brandon M. P. Koenig J. Leutgeb J. K. Leutgeb S. (2014). New and distinct hippocampal place codes are generated in a new environment during septal inactivation. Neuron 82, 789–796. 10.1016/j.neuron.2014.04.013 24853939 PMC4294702

[B6] Brauer K. Seeger G. Hartig W. Rossner S. Poethke R. Kacza J.. (1998). Electron microscopic evidence for a cholinergic innervation of GABAergic parvalbumin-immunoreactive neurons in the rat medial septum. J. Neurosci. Res. 54, 248–253. 9788283 10.1002/(SICI)1097-4547(19981015)54:2<248::AID-JNR12>3.0.CO;2-0

[B7] Brun V. H. Otnaess M. K. Molden S. Steffenach H. A. Witter M. P. Moser M. B.. (2002). Place cells and place recognition maintained by direct entorhinal-hippocampal circuitry. Science 296, 2243–2246. 10.1126/science.1071089 12077421

[B8] Buzsaki G. Leung L. W. Vanderwolf C. H. (1983). Cellular bases of hippocampal EEG in the behaving rat. Brain Res. 287, 139–171. 10.1016/0165-0173(83)90037-1 6357356

[B9] Calton J. L. Stackman R. W. Goodridge J. P. Archey W. B. Dudchenko P. A. Taube J. S. (2003). Hippocampal place cell instability after lesions of the head direction cell network. J. Neurosci. 23, 9719–9731. 14585999 10.1523/JNEUROSCI.23-30-09719.2003 PMC6740880

[B10] Canolty R. T. Ganguly K. Kennerley S. W. Cadieu C. F. Koepsell K. Wallis J. D.. (2010). Oscillatory phase coupling coordinates anatomically dispersed functional cell assemblies. Proc. Natl. Acad. Sci. U.S.A. 107, 17356–17361. 10.1073/pnas.1008306107 20855620 PMC2951408

[B11] Cardin J. A. Carlen M. Meletis K. Knoblich U. Zhang F. Deisseroth K.. (2010). Targeted optogenetic stimulation and recording of neurons *in vivo* using cell-type-specific expression of Channelrhodopsin-2. Nat. Protoc. 5, 247–254. 10.1038/nprot.2009.228 20134425 PMC3655719

[B12] Chapman C. A. Lacaille J. C. (1999). Cholinergic induction of theta-frequency oscillations in hippocampal inhibitory interneurons and pacing of pyramidal cell firing. J. Neurosci. 19, 8637–8645. 10493764 10.1523/JNEUROSCI.19-19-08637.1999 PMC6783040

[B13] Colom L. V. Castaneda M. T. Reyna T. Hernandez S. Garrido-Sanabria E. (2005). Characterization of medial septal glutamatergic neurons and their projection to the hippocampus. Synapse 58, 151–164. 10.1002/syn.20184 16108008

[B14] Csicsvari J. Hirase H. Czurko A. Mamiya A. Buzsaki G. (1999). Oscillatory coupling of hippocampal pyramidal cells and interneurons in the behaving Rat. J. Neurosci. 19, 274–287. 9870957 10.1523/JNEUROSCI.19-01-00274.1999 PMC6782375

[B15] Dragoi G. Carpi D. Recce M. Csicsvari J. Buzsaki G. (1999). Interactions between hippocampus and medial septum during sharp waves and theta oscillation in the behaving rat. J. Neurosci. 19, 6191–6199. 10407055 10.1523/JNEUROSCI.19-14-06191.1999 PMC6783073

[B16] Dragoi G. Harris K. D. Buzsáki G. (2003). Place representation within hippocampal networks is modified by long-term potentiation. Neuron 39, 843–853. 10.1016/S0896-6273(03)00465-3 12948450

[B17] Fking C. Recce M. O'keefe J. (1998). The rhythmicity of cells of the medial septum/diagonal band of Broca in the awake freely moving rat: relationships with behaviour and hippocampal theta. Eur. J. Neurosci. 10, 464–477. 10.1046/j.1460-9568.1998.00026.x 9749709

[B18] Freund T. F. Gulyas A. I. (1997). Inhibitory control of GABAergic interneurons in the hippocampus. Can. J. Physiol. Pharmacol. 75, 479–487. 10.1139/y97-033 9250381

[B19] Gritti I. Henny P. Galloni F. Mainville L. Mariotti M. Jones B. E. (2006). Stereological estimates of the basal forebrain cell population in the rat, including neurons containing choline acetyltransferase, glutamic acid decarboxylase or phosphate-activated glutaminase and colocalizing vesicular glutamate transporters. Neuroscience 143, 1051–1064. 10.1016/j.neuroscience.2006.09.024 17084984 PMC1831828

[B20] Hok V. Chah E. Save E. Poucet B. (2013). Prefrontal cortex focally modulates hippocampal place cell firing patterns. J. Neurosci. 33, 3443–3451. 10.1523/JNEUROSCI.3427-12.2013 23426672 PMC6619543

[B21] Hollup S. A. Molden S. Donnett J. G. Moser M. B. Moser E. I. (2001). Place fields of rat hippocampal pyramidal cells and spatial learning in the watermaze. Eur. J. Neurosci. 13, 1197–1208. 10.1046/j.0953-816x.2001.01487.x 11285017

[B22] Huh C. Y. Goutagny R. Williams S. (2010). Glutamatergic neurons of the mouse medial septum and diagonal band of Broca synaptically drive hippocampal pyramidal cells: relevance for hippocampal theta rhythm. J. Neurosci. 30, 15951–15961. 10.1523/JNEUROSCI.3663-10.2010 21106833 PMC6633737

[B23] Jones B. E. (2005). From waking to sleeping: neuronal and chemical substrates. Trends Pharmacol. Sci. 26, 578–586. 10.1016/j.tips.2005.09.009 16183137

[B24] Kaifosh P. Lovett-Barron M. Turi G. F. Reardon T. R. Losonczy A. (2013). Septo-hippocampal GABAergic signaling across multiple modalities in awake mice. Nat. Neurosci. 16, 1182–1184. 10.1038/nn.3482 23912949

[B25] Koenig J. Linder A. N. Leutgeb J. K. Leutgeb S. (2011). The spatial periodicity of grid cells is not sustained during reduced theta oscillations. Science 332, 592–595. 10.1126/science.1201685 21527713

[B26] Lachaux J. P. Rodriguez E. Martinerie J. Varela F. J. (1999). Measuring phase synchrony in brain signals. Hum. Brain Mapp. 8, 194–208. 10619414 10.1002/(SICI)1097-0193(1999)8:4<194::AID-HBM4>3.0.CO;2-C PMC6873296

[B27] Lee M. G. Chrobak J. J. Sik A. Wiley R. G. Buzsaki G. (1994). Hippocampal theta activity following selective lesion of the septal cholinergic system. Neuroscience 62, 1033–1047. 10.1016/0306-4522(94)90341-7 7845584

[B28] Lee S. H. Dan Y. (2012). Neuromodulation of brain states. Neuron 76, 209–222. 10.1016/j.neuron.2012.09.012 23040816 PMC3579548

[B29] Leranth C. Deller T. Buzsaki G. (1992). Intraseptal connections redefined: lack of a lateral septum to medial septum path. Brain Res. 583, 1–11. 10.1016/S0006-8993(10)80004-6 1380395

[B30] Leutgeb S. Mizumori S. J. (1999). Excitotoxic septal lesions result in spatial memory deficits and altered flexibility of hippocampal single-unit representations. J. Neurosci. 19, 6661–6672. 10414995 10.1523/JNEUROSCI.19-15-06661.1999 PMC6782835

[B31] Lovett-Barron M. Kaifosh P. Kheirbek M. A. Danielson N. Zaremba J. D. Reardon T. R.. (2014). Dendritic inhibition in the hippocampus supports fear learning. Science 343, 857–863. 10.1126/science.1247485 24558155 PMC4018419

[B32] Lovett-Barron M. Losonczy A. (2014). Behavioral consequences of GABAergic neuronal diversity. Curr. Opin. Neurobiol. 26, 27–33. 10.1016/j.conb.2013.11.002 24650501

[B33] Manns I. D. Mainville L. Jones B. E. (2001). Evidence for glutamate, in addition to acetylcholine and GABA, neurotransmitter synthesis in basal forebrain neurons projecting to the entorhinal cortex. Neuroscience 107, 249–263. 10.1016/S0306-4522(01)00302-5 11731099

[B34] Manseau F. Danik M. Williams S. (2005). A functional glutamatergic neurone network in the medial septum and diagonal band area. J. Physiol. 566, 865–884. 10.1113/jphysiol.2005.089664 15919710 PMC1464770

[B35] Marrosu F. Portas C. Mascia M. S. Casu M. A. Fa M. Giagheddu M.. (1995). Microdialysis measurement of cortical and hippocampal acetylcholine release during sleep-wake cycle in freely moving cats. Brain Res. 671, 329–332. 10.1016/0006-8993(94)01399-3 7743225

[B36] Muller R. U. Kubie J. L. (1989). The firing of hippocampal place cells predicts the future position of freely moving rats. J. Neurosci. 9, 4101–4110. 2592993 10.1523/JNEUROSCI.09-12-04101.1989 PMC6569642

[B37] Nagel G. Szellas T. Huhn W. Kateriya S. Adeishvili N. Berthold P.. (2003). Channelrhodopsin-2, a directly light-gated cation-selective membrane channel. Proc. Natl. Acad. Sci. U.S.A. 100, 13940–13945. 10.1073/pnas.1936192100 14615590 PMC283525

[B38] Oddie S. D. Stefanek W. Kirk I. J. Bland B. H. (1996). Intraseptal procaine abolishes hypothalamic stimulation-induced wheel-running and hippocampal theta field activity in rats. J. Neurosci. 16, 1948–1956. 8774461 10.1523/JNEUROSCI.16-05-01948.1996 PMC6578689

[B39] O'keefe J. (1976). Place units in the hippocampus of the freely moving rat. Exp. Neurol. 51, 78–109. 10.1016/0014-4886(76)90055-8 1261644

[B40] O'keefe J. Recce M. L. (1993). Phase relationship between hippocampal place units and the EEG theta rhythm. Hippocampus 3, 317–330. 10.1002/hipo.450030307 8353611

[B41] Parikh V. Kozak R. Martinez V. Sarter M. (2007). Prefrontal acetylcholine release controls cue detection on multiple timescales. Neuron 56, 141–154. 10.1016/j.neuron.2007.08.025 17920021 PMC2084212

[B42] Pastalkova E. Itskov V. Amarasingham A. Buzsaki G. (2008). Internally generated cell assembly sequences in the rat hippocampus. Science 321, 1322–1327. 10.1126/science.1159775 18772431 PMC2570043

[B43] Poucet B. Herrmann T. Buhot M. C. (1991). Effects of short-lasting inactivations of the ventral hippocampus and medial septum on long-term and short-term acquisition of spatial information in rats. Behav. Brain Res. 44, 53–65. 10.1016/S0166-4328(05)80239-6 1910571

[B44] Quirk G. J. Muller R. U. Kubie J. L. (1990). The firing of hippocampal place cells in the dark depends on the rat's recent experience. J. Neurosci. 10, 2008–2017. 2355262 10.1523/JNEUROSCI.10-06-02008.1990 PMC6570323

[B45] Rawlins J. N. Feldon J. Gray J. A. (1979). Septo-hippocampal connections and the hippocampal theta rhythm. Exp. Brain Res. 37, 49–63. 10.1007/BF01474253 385334

[B46] Robbe D. Buzsaki G. (2009). Alteration of theta timescale dynamics of hippocampal place cells by a cannabinoid is associated with memory impairment. J. Neurosci. 29, 12597–12605. 10.1523/JNEUROSCI.2407-09.2009 19812334 PMC2799373

[B47] Scarlett D. Dypvik A. T. Bland B. H. (2004). Comparison of spontaneous and septally driven hippocampal theta field and theta-related cellular activity. Hippocampus 14, 99–106. 10.1002/hipo.10151 15058487

[B48] Sharp P. E. Green C. (1994). Spatial correlates of firing patterns of single cells in the subiculum of the freely moving rat. J. Neurosci. 14, 2339–2356. 8158272 10.1523/JNEUROSCI.14-04-02339.1994 PMC6577112

[B49] Simon A. P. Poindessous-Jazat F. Dutar P. Epelbaum J. Bassant M. H. (2006). Firing properties of anatomically identified neurons in the medial septum of anesthetized and unanesthetized restrained rats. J. Neurosci. 26, 9038–9046. 10.1523/JNEUROSCI.1401-06.2006 16943562 PMC6675331

[B50] Skaggs W. E. Mcnaughton B. L. Gothard K. M. (1993). An Information-theoretic Approach to Deciphering the Hippocampal Code. San Francisco, CA: Morgan Kaufmann Publishers Inc.

[B51] Skaggs W. E. Mcnaughton B. L. Wilson M. A. Barnes C. A. (1996). Theta phase precession in hippocampal neuronal populations and the compression of temporal sequences. Hippocampus 6, 149–172. 8797016 10.1002/(SICI)1098-1063(1996)6:2<149::AID-HIPO6>3.0.CO;2-K

[B52] Sotty F. Danik M. Manseau F. Laplante F. Quirion R. Williams S. (2003). Distinct electrophysiological properties of glutamatergic, cholinergic and GABAergic rat septohippocampal neurons: novel implications for hippocampal rhythmicity. J. Physiol. 551, 927–943. 10.1113/jphysiol.2003.046847 12865506 PMC2343277

[B53] Teles-Grilo Ruivo L. M. Mellor J. R. (2013). Cholinergic modulation of hippocampal network function. Front. Synaptic Neurosci. 5:2. 10.3389/fnsyn.2013.00002 23908628 PMC3726829

[B54] Toth K. Freund T. F. Miles R. (1997). Disinhibition of rat hippocampal pyramidal cells by GABAergic afferents from the septum. J. Physiol. 500 (Pt 2), 463–474. 10.1113/jphysiol.1997.sp022033 9147330 PMC1159396

[B54a] Tsai H. C. Zhang F. Adamantidis A. Stuber G. D. Bonci A. De Lecea L. Deisseroth K. (2009). Phasic firing in dopaminergic neurons is sufficient for behavioral conditioning. Science 324, 1080–1084. 10.1126/science.1168878 19389999 PMC5262197

[B55] Tsanov M. Chah E. Reilly R. O'mara S. M. (2014). Respiratory cycle entrainment of septal neurons mediates the fast coupling of sniffing rate and hippocampal theta rhythm. Eur. J. Neurosci. 39, 957–974. 10.1111/ejn.12449 24329896 PMC4165309

[B56] Tsanov M. Chah E. Vann S. D. Reilly R. B. Erichsen J. T. Aggleton J. P.. (2011a). Theta-modulated head direction cells in the rat anterior thalamus. J. Neurosci. 31, 9489–9502. 10.1523/JNEUROSCI.0353-11.2011 21715614 PMC3855197

[B57] Tsanov M. Chah E. Wright N. Vann S. D. Reilly R. Erichsen J. T.. (2011b). Oscillatory entrainment of thalamic neurons by theta rhythm in freely moving rats. J. Neurophysiol. 105, 4–17. 10.1152/jn.00771.2010 20962067 PMC3023377

[B58] Tsao A. Moser M. B. Moser E. I. (2013). Traces of experience in the lateral entorhinal cortex. Curr. Biol. 23, 399–405. 10.1016/j.cub.2013.01.036 23434282

[B59] Vandecasteele M. Varga V. Berenyi A. Papp E. Bartho P. Venance L.. (2014). Optogenetic activation of septal cholinergic neurons suppresses sharp wave ripples and enhances theta oscillations in the hippocampus. Proc. Natl. Acad. Sci. U.S.A. 111, 13535–13540. 10.1073/pnas.1411233111 25197052 PMC4169920

[B60] Vertes R. P. Kocsis B. (1997). Brainstem-diencephalo-septohippocampal systems controlling the theta rhythm of the hippocampus. Neuroscience 81, 893–926. 9330355 10.1016/s0306-4522(97)00239-x

[B61] Vinogradova O. S. (1995). Expression, control, and probable functional significance of the neuronal theta-rhythm. Prog. Neurobiol. 45, 523–583. 10.1016/0301-0082(94)00051-I 7624485

[B62] Wang Y. Romani S. Lustig B. Leonardo A. Pastalkova E. (2015). Theta sequences are essential for internally generated hippocampal firing fields. Nat. Neurosci. 18, 282–288. 10.1038/nn.3904 25531571

[B63] Widmer H. Ferrigan L. Davies C. H. Cobb S. R. (2006). Evoked slow muscarinic acetylcholinergic synaptic potentials in rat hippocampal interneurons. Hippocampus 16, 617–628. 10.1002/hipo.20191 16770798

[B64] Williams J. M. Givens B. (2003). Stimulation-induced reset of hippocampal theta in the freely performing rat. Hippocampus 13, 109–116. 10.1002/hipo.10082 12625462

[B65] Wilson M. A. Mcnaughton B. L. (1993). Dynamics of the hippocampal ensemble code for space. Science 261, 1055–1058. 10.1126/science.8351520 8351520

[B66] Winson J. (1978). Loss of hippocampal theta rhythm results in spatial memory deficit in the rat. Science 201, 160–163. 10.1126/science.663646 663646

[B67] Witten I. B. Steinberg E. E. Lee S. Y. Davidson T. J. Zalocusky K. A. Brodsky M.. (2011). Recombinase-driver rat lines: tools, techniques, and optogenetic application to dopamine-mediated reinforcement. Neuron 72, 721–733. 10.1016/j.neuron.2011.10.028 22153370 PMC3282061

[B68] Yizhar O. Fenno L. E. Davidson T. J. Mogri M. Deisseroth K. (2011). Optogenetics in neural systems. Neuron 71, 9–34. 10.1016/j.neuron.2011.06.004 21745635

[B69] Yoder R. M. Pang K. C. (2005). Involvement of GABAergic and cholinergic medial septal neurons in hippocampal theta rhythm. Hippocampus 15, 381–392. 10.1002/hipo.20062 15630696

[B70] Zhang H. Lin S. C. Nicolelis M. A. (2010). Spatiotemporal coupling between hippocampal acetylcholine release and theta oscillations *in vivo*. J. Neurosci. 30, 13431–13440. 10.1523/JNEUROSCI.1144-10.2010 20926669 PMC2988451

[B71] Zhang H. Lin S. C. Nicolelis M. A. (2011). A distinctive subpopulation of medial septal slow-firing neurons promote hippocampal activation and theta oscillations. J. Neurophysiol. 106, 2749–2763. 10.1152/jn.00267.2011 21865435 PMC3214118

