# Supplementary material for: Medial septum regulates the hippocampal spatial representation
Source: Front Behav Neurosci. 2015 Jun 30;9:166. doi: 10.3389/fnbeh.2015.00166 (PMC4485312; doi:10.3389/fnbeh.2015.00166)
Supplement: Supplementary file 1 [file DataSheet1.DOCX]

***Supplementary Material***

**Medial septum regulates the hippocampal spatial representation**

Omar Mamad^1,2^, Harold M. McNamara^1,3^ Richard Reilly^1,3^ and Marian Tsanov ^1,2,^*

^1^ Trinity College Institute of Neuroscience, Trinity College Dublin, Ireland

^2^ School of Psychology, Trinity College Dublin, Ireland.

^3^Trinity Centre for Bioengineering, Trinity College Dublin, Ireland.

***Correspondence:** Marian Tsanov:

Trinity College Institute of Neuroscience, Trinity College Dublin, Dublin 2, Ireland

Fax: +353-1-896 3183; Tel: +353-1-896 4829; E-mail: [tsanovm@tcd.ie](mailto:tsanovm@tcd.ie)

**Supplementary Figures**

**Supplementary Figure 1:**

**
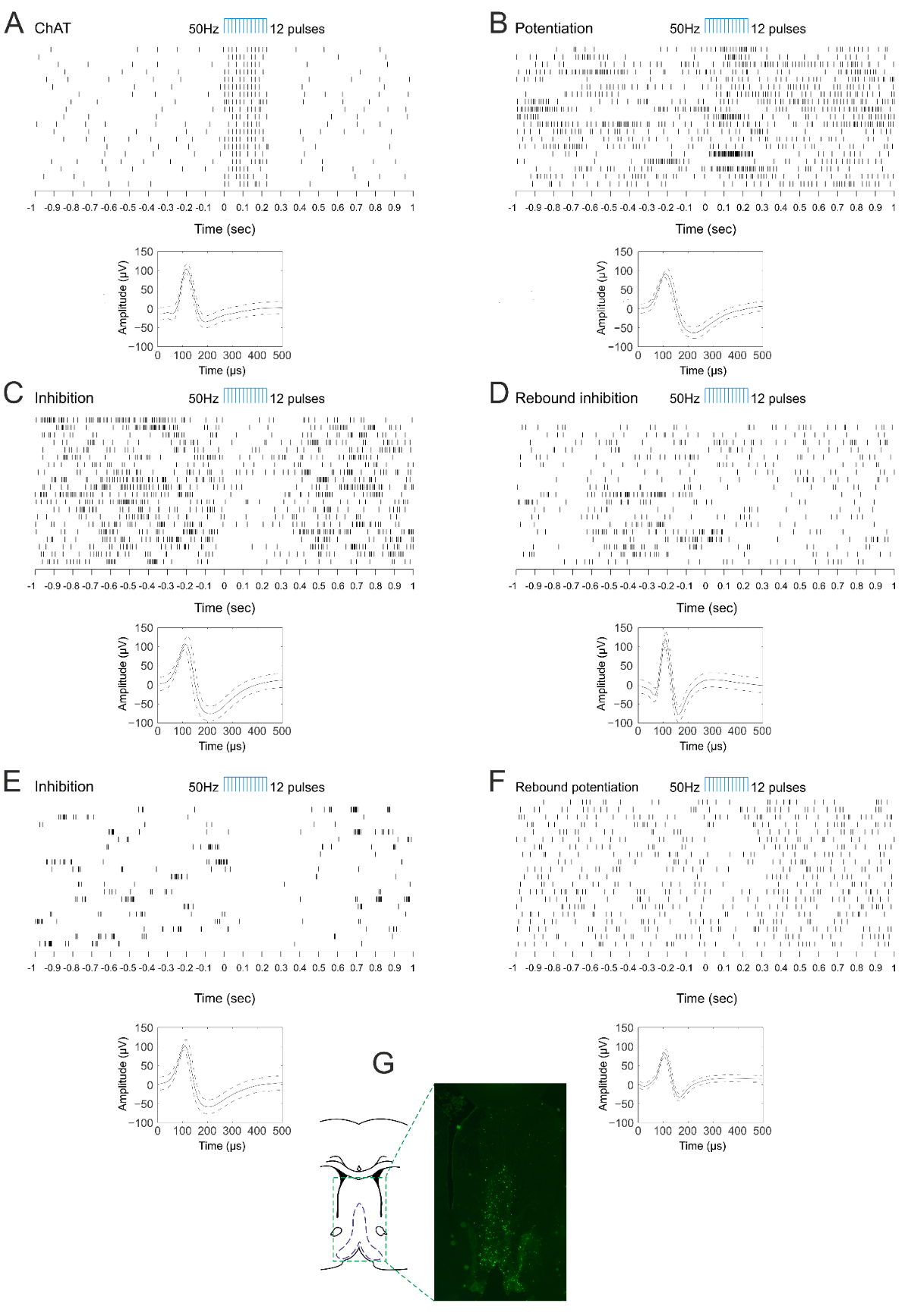
**

**Supplementary Figure 1.** Neuronal responses after optogenetic stimulation in medial septum. Raster plot of representative neuronal responses from ChAT (A), potentiation (B), fast-spiking inhibition (C), re-inhibition (D), slow-spiking inhibition (E) and re-potentiation (F) cells in medial septum. The recording interval ranges from 1 sec before and 1 s after the stimulation onset. Bottom: spike waveforms of the recorded cells. (G) Coronal atlas schematic and histological section (right) showing the degree of ChR2-YFP expression in medial septum.

**Supplementary Figure 2:**

**
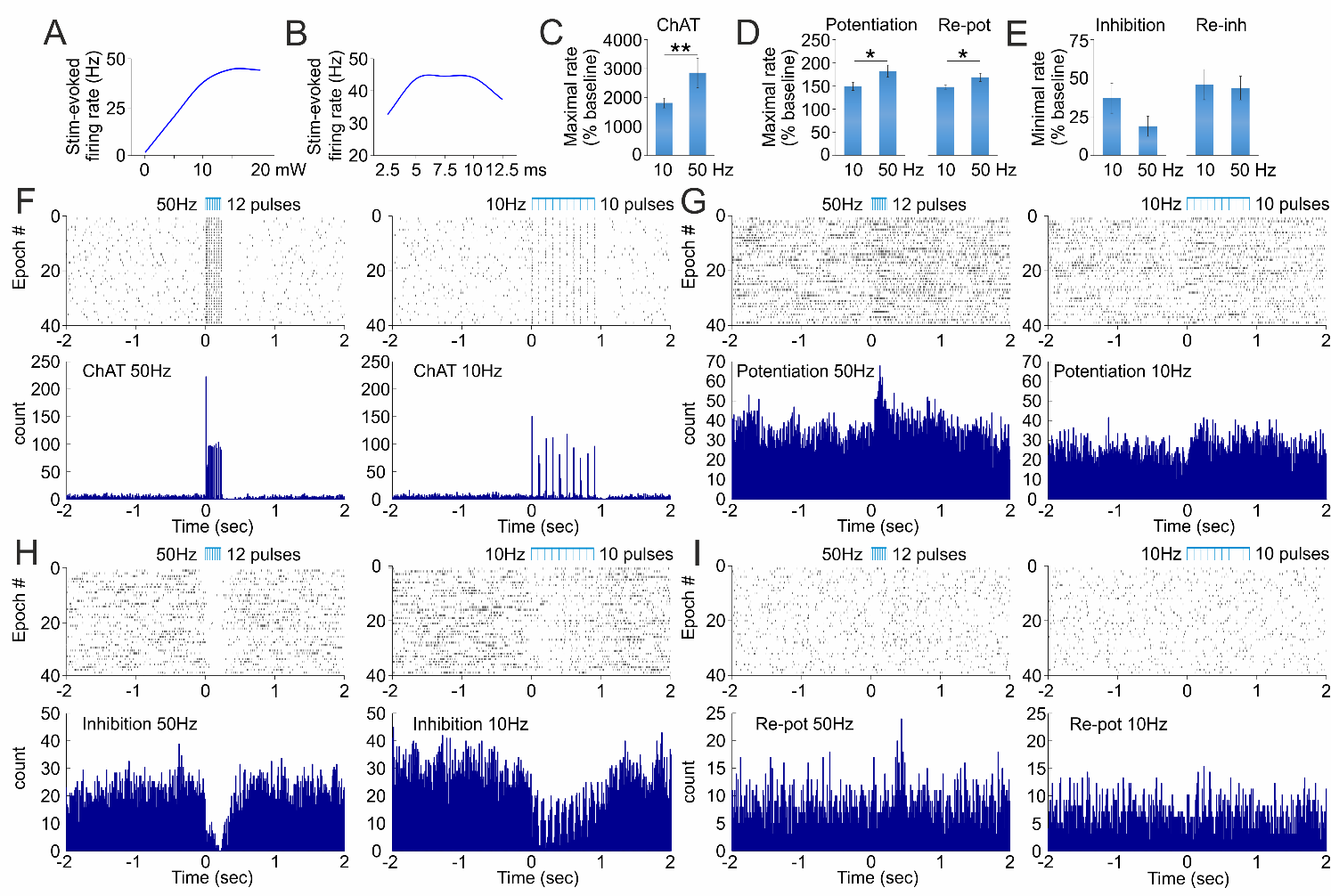
**

**Supplementary Figure 2.** Dependence of the septal network response on the optogenetic stimulation parameters. Firing rate of light-entrained ChAT neurons as a function of the laser light power (A) and pulse duration (B). Comparison between the stimulation-evoked maximal firing rate (percent of baseline) between 10 and 50 Hz stimulation for the neurons from the ChAT (C), potentiation and re-potentiation (D) groups. (E) Comparison between the stimulation-evoked minimal firing rate (percent of baseline) between 10 and 50 Hz stimulation for the neurons from the inhibition and re-inhibition groups. Error bars represent ± sem, paired *t*-test; *P < 0.05, **P < 0.01. (F) Raster plot from 40 repetitions (above) and spike count of 120 repetitions (below) of optically evoked time-locked responses of representative ChAT cell after 50 (left) and 10 Hz (right) stimulation protocol. (G) Raster plot and of representative potentiation cell after 50 (left) and 10 Hz (right) Hz stimulation protocol. (H) Raster plot and spike count of representative inhibition cell after 50 (left) and 10 Hz (right) stimulation protocol. (I) Raster plot and spike count of representative re-potentiation cell after 50 (left) and 10 Hz (right) stimulation protocol.

**Supplementary Figure 3:**


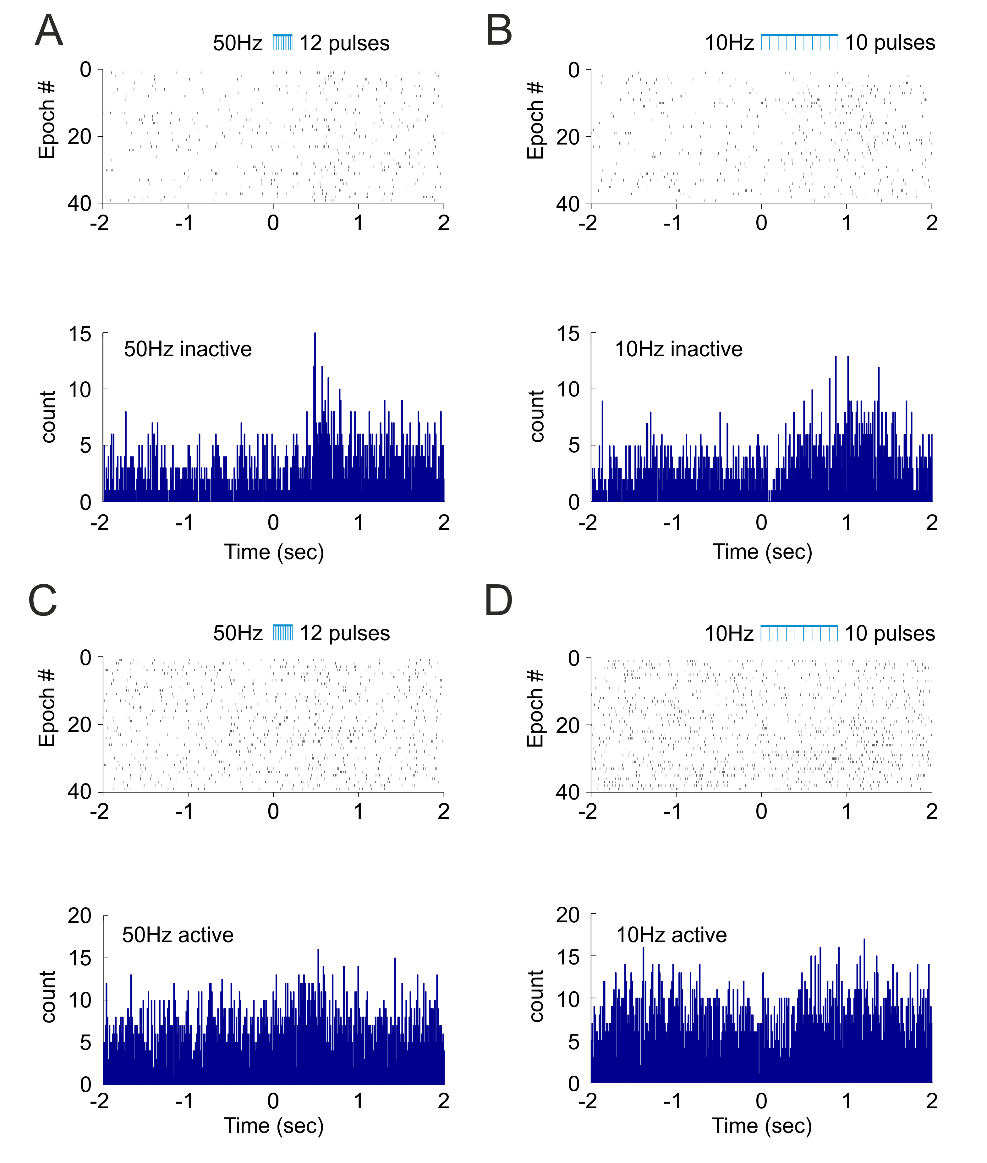


**Supplementary Figure 3**. Dependence of the hippocampal neuronal response on the behavioral state. Raster plot from 40 repetitions (above) and spike count of 120 repetitions (below) of representative hippocampal interneuron during inactive behavioural state after 50 Hz (A) and 10 Hz (B) stimulation protocol. (C) Raster plot and spike count of the same cell during active behavioural state after 50 Hz (C) and 10 Hz (D) stimulation protocol. Time 0 indicates the delivery of the first train of the stimulation protocol to medial septum.

**Supplementary Figure 4:**

**
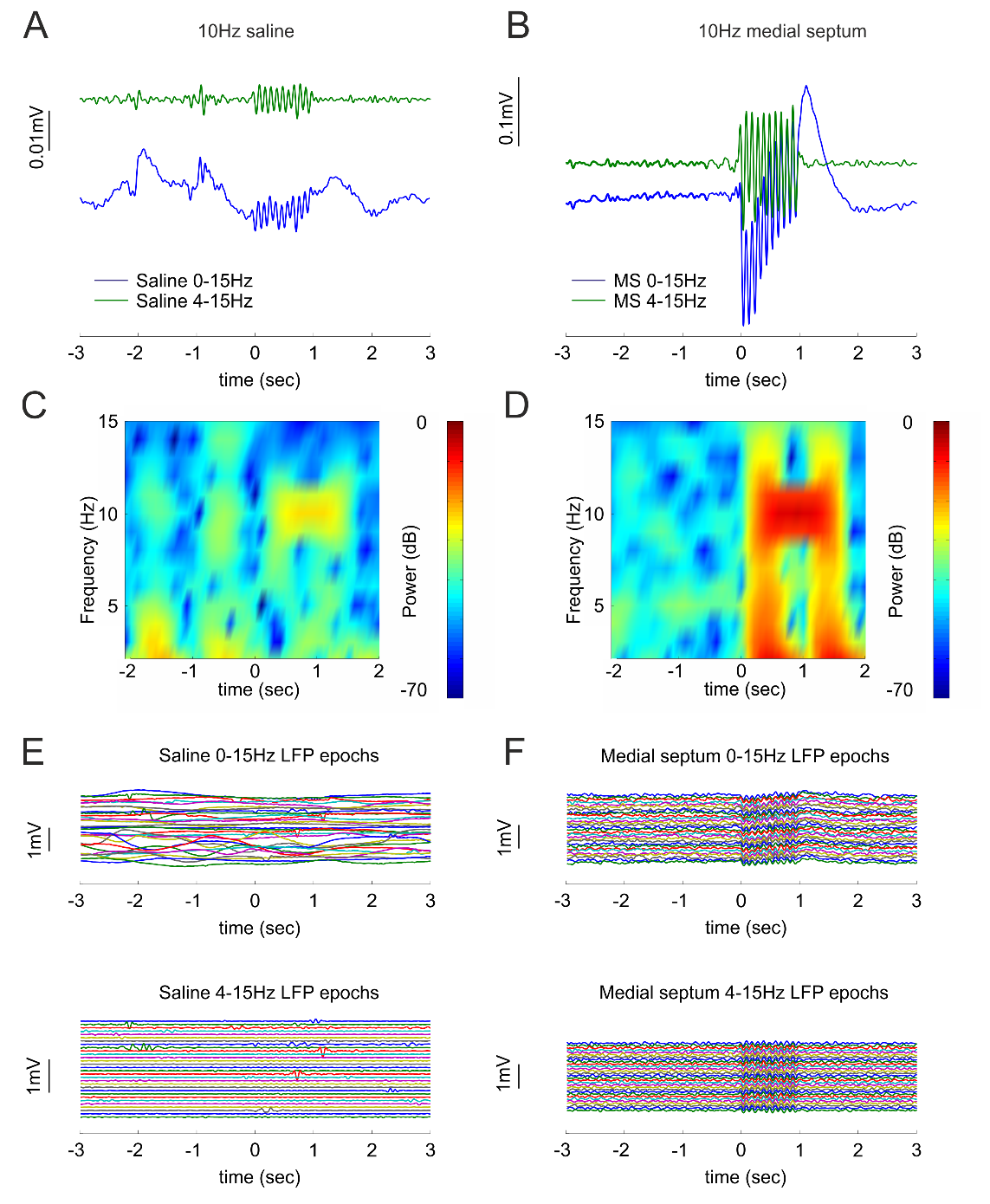
**

**Supplementary Figure 4**. Photoelectric effect in medial septum. Event related potentials (ERP) recorded in saline 0.9% (A) and in medial septum (B) after application of blue laser light. Upper green traces show are band-pass filtered (4 – 15 Hz), while lower blue traces are low-pass filtered (0 – 15 Hz). Time 0 indicates the delivery of the first train of 10Hz laser light stimulation. Color-coded power spectrograms of low-frequency oscillations after 10 Hz stimulation in saline 0.9% (C) and in medial septum (D) after application of blue laser light. Individual traces of local field potentials recorded from 32 electrodes in saline (E) and medial septum (F) after low-pass filtered (above, 0 – 15 Hz) and band-pass (below, 4 – 15 Hz) filters.

**Supplementary Figure 5:**

**
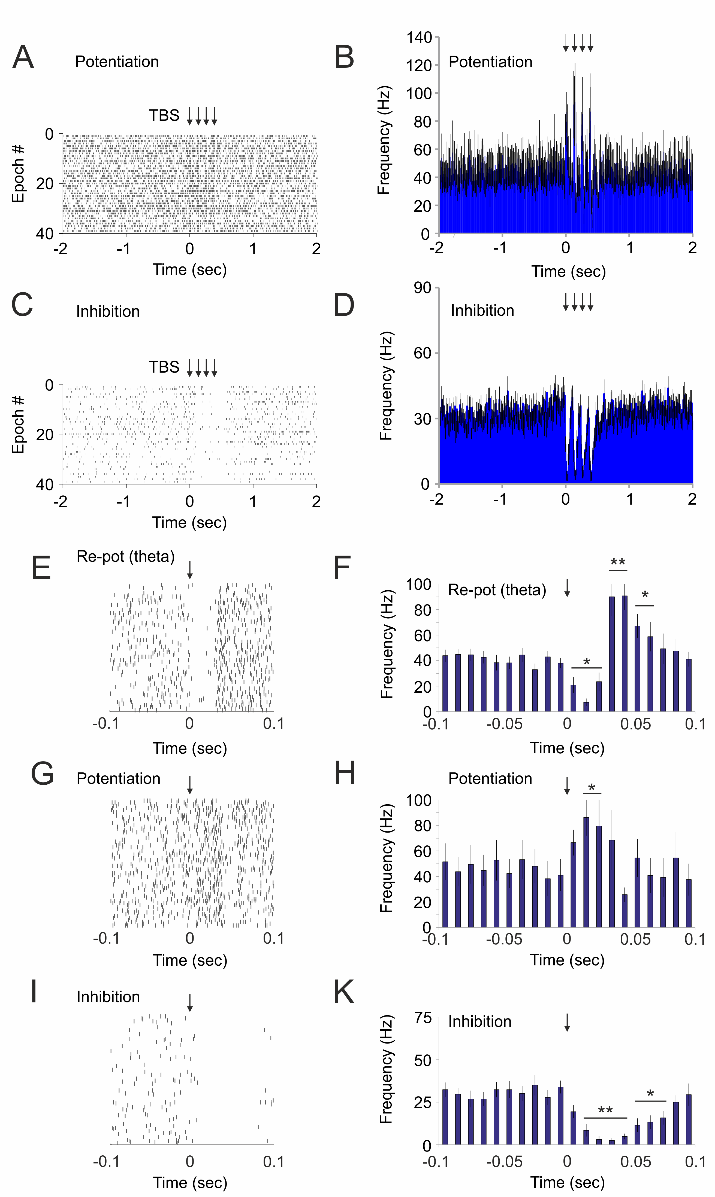
**

**Supplementary Figure 5**. Hippocampal neuronal differential response after septal theta-burst stimulation. (A) Raster plot from 40 repetitions (above) and spike count of 120 repetitions (below) of representative potentiation hippocampal cell after electric theta-burst stimulation protocol (TBS, 4 bursts with inter-train interval of 100 Hz and inter-train interval of 8Hz) to medial septum. Time 0 indicates the delivery of the first train. The vertical arrows indicate the delivery time of four trains. (B) Averaged frequency histogram for all potentiation cells. The vertical arrows indicate the delivery time of four trains. Black error bars represent ± sem. (C) Raster plot and spike count of representative inhibition cell after TBS to medial septum. (D) Averaged frequency histogram for all inhibition cells. Black error bars represent ± sem. (E) Raster plot of representative re-potentiation hippocampal cell after the first train of TBS to medial septum. The recording interval ranges from -0.1 ms before and 0.1 ms after the first train. The vertical arrow indicates the delivery time of the first train. (F) Spiking histogram of the averaged firing frequency from -0.1 ms before and 0.1 ms after the first train for the re-potentiated (theta) units. (G) Raster plot of representative potentiation hippocampal cell after the first train of TBS to medial septum. (H) Spiking histogram of the averaged firing frequency from -0.1 ms before and 0.1 ms after the first train for the potentiation units. (I) Raster plot of representative inhibition hippocampal cell after the first train of TBS to medial septum. (K) Spiking histogram of the averaged firing frequency from -0.1 ms before and 0.1 ms after the first train for the inhibition units. Error bars represent ± sem, two-way ANOVA, *P < 0.05.

**Supplementary Figure 6:**


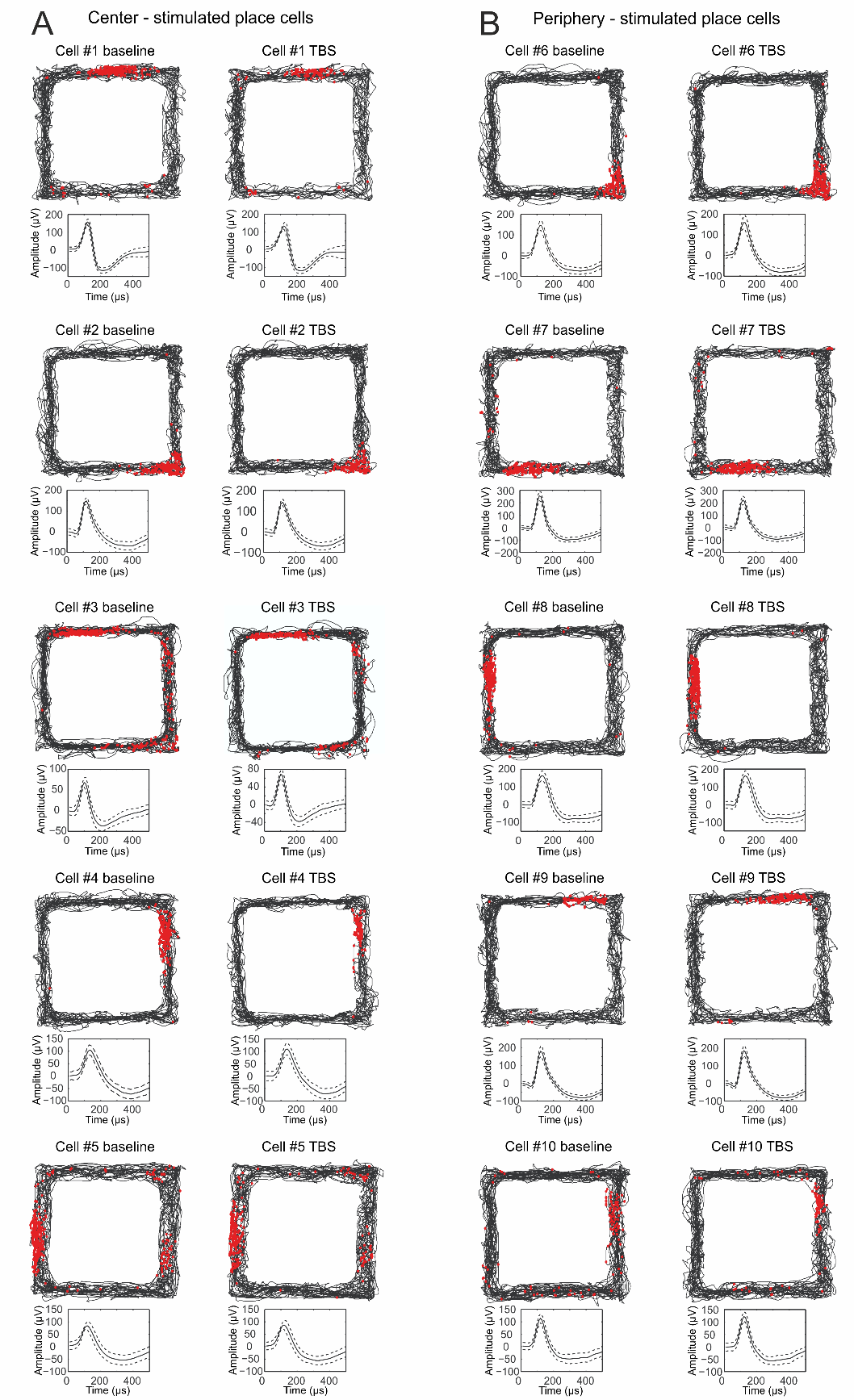


**Supplementary Figure 6.** Differential theta-burst stimulation effect on place cells. Sample place field maps of CA1 pyramidal neurons from five center-stimulated (A) and five periphery-stimulated (B) place cells. The map represents animal trajectory with spikes shown in red. Left panels show the baseline session, while right panels show the stimulation session. Bottom: spike waveforms of the recorded place cells during the baseline and the stimulation sessions.
