# Supplementary figures and images for: Medial septum regulates the hippocampal spatial representation
Source: Front Behav Neurosci. 2015 Jun 30;9:166. doi: 10.3389/fnbeh.2015.00166 (PMC4485312; doi:10.3389/fnbeh.2015.00166)

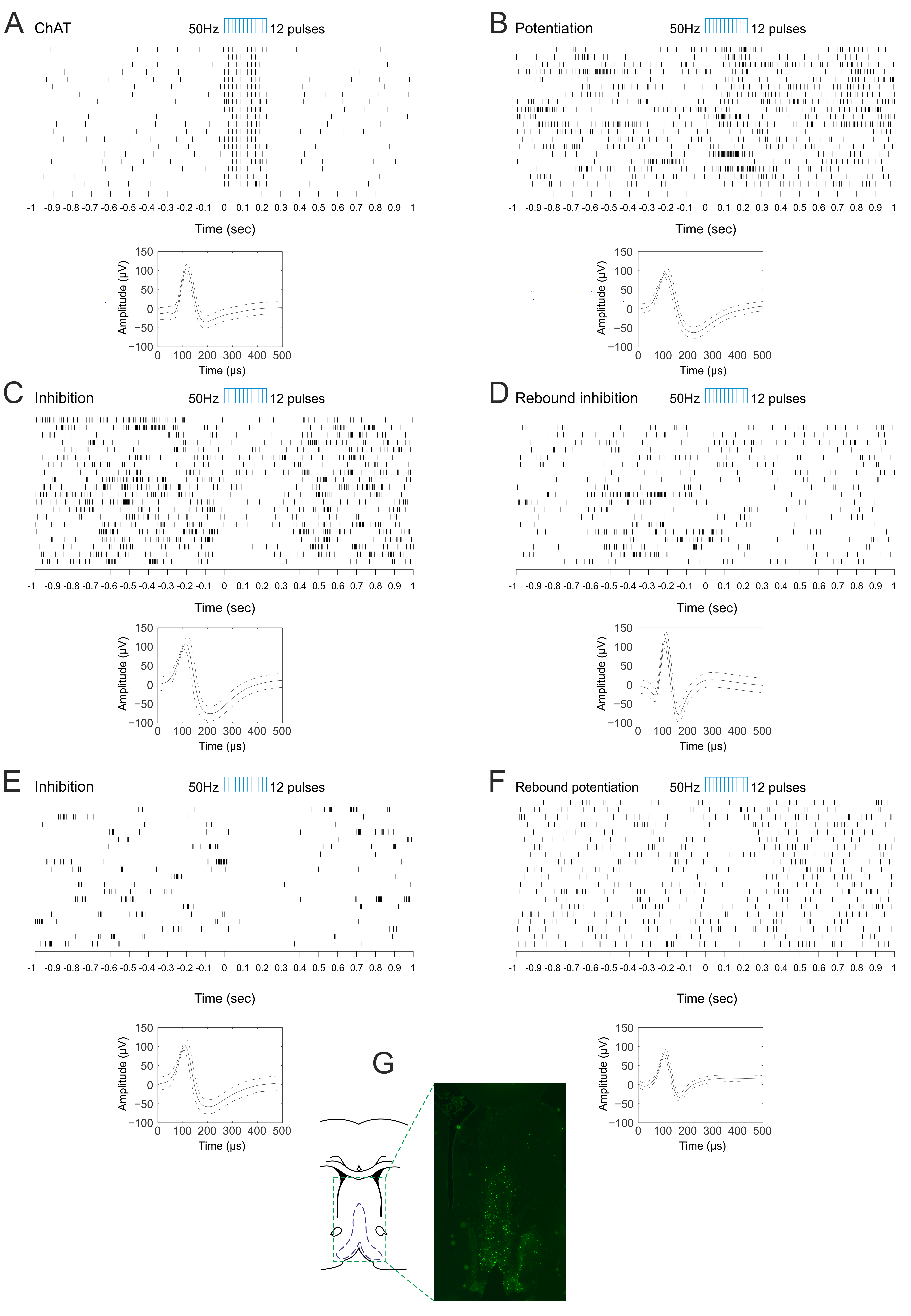

Supplement: Supplementary file 2 [file Image1.TIF]

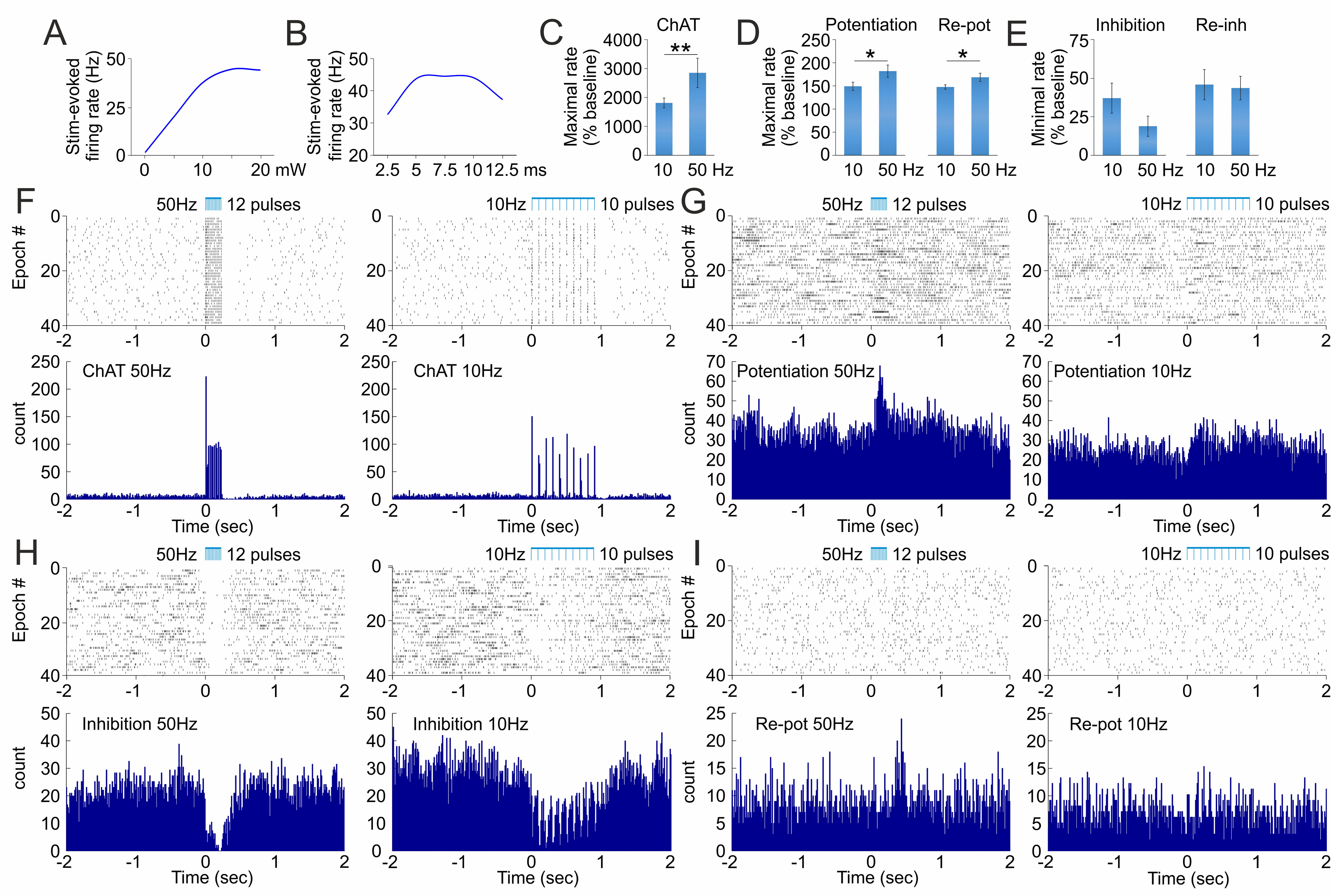

Supplement: Supplementary file 3 [file Image2.TIF]

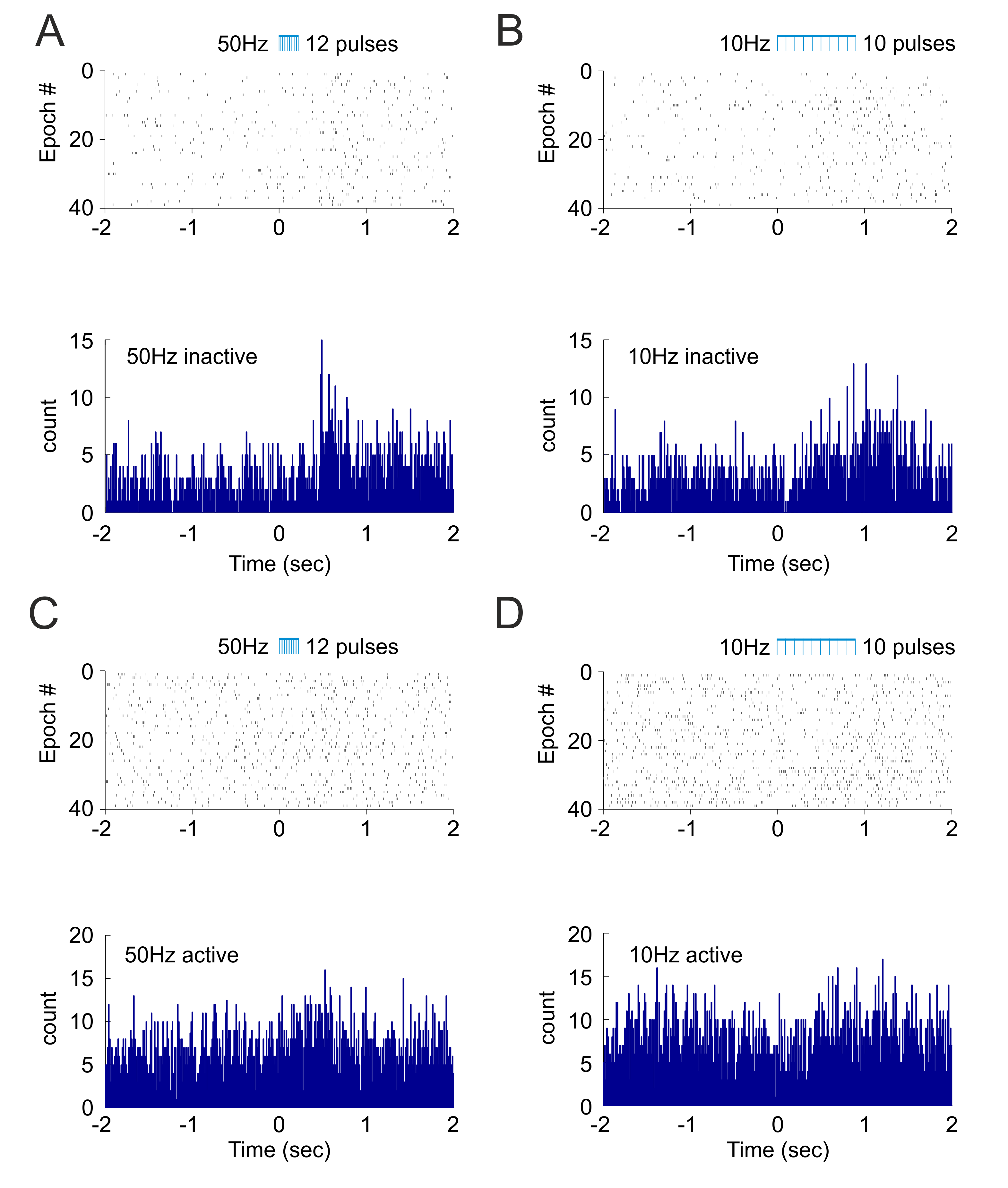

Supplement: Supplementary file 4 [file Image3.TIF]

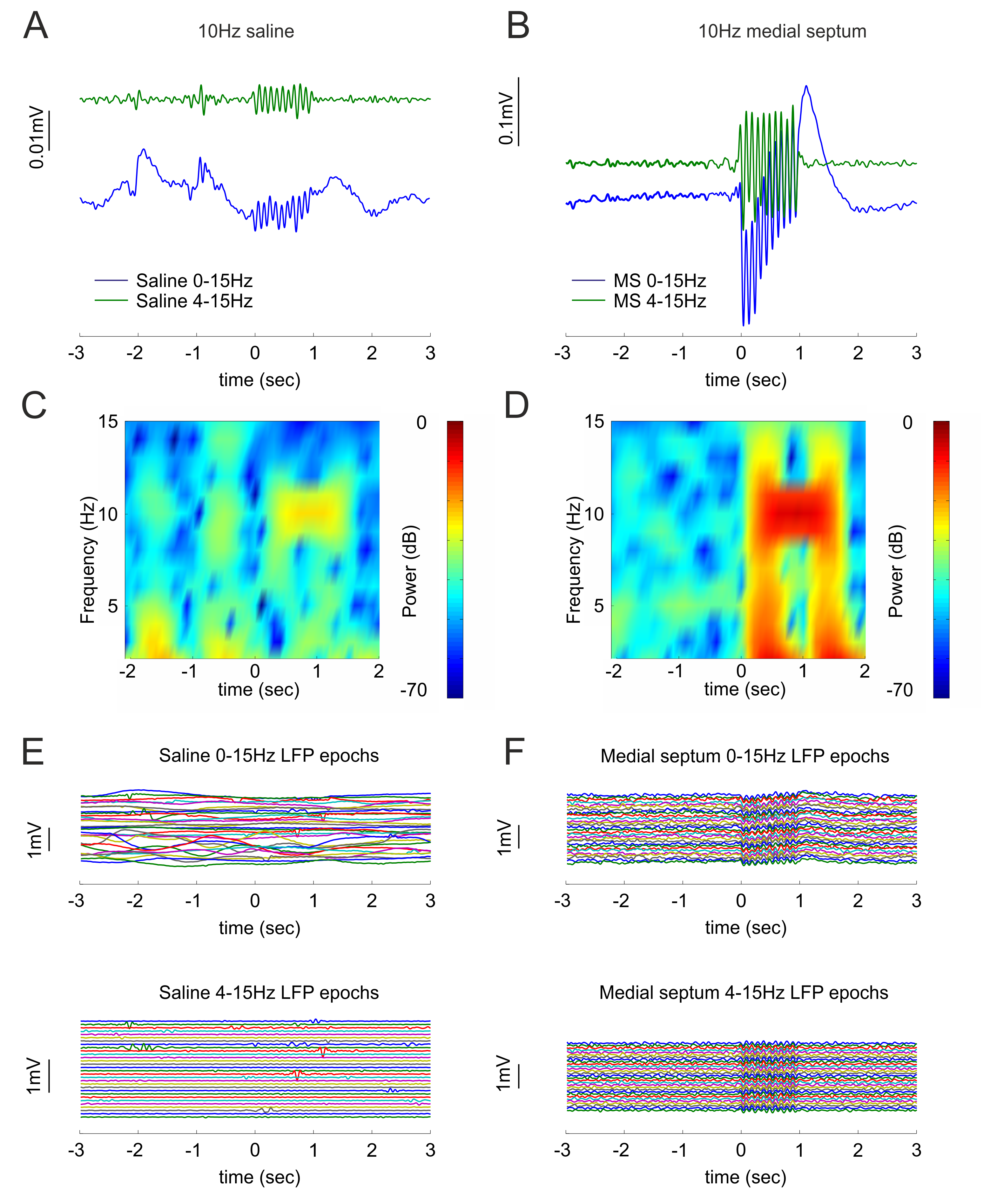

Supplement: Supplementary file 5 [file Image4.TIF]

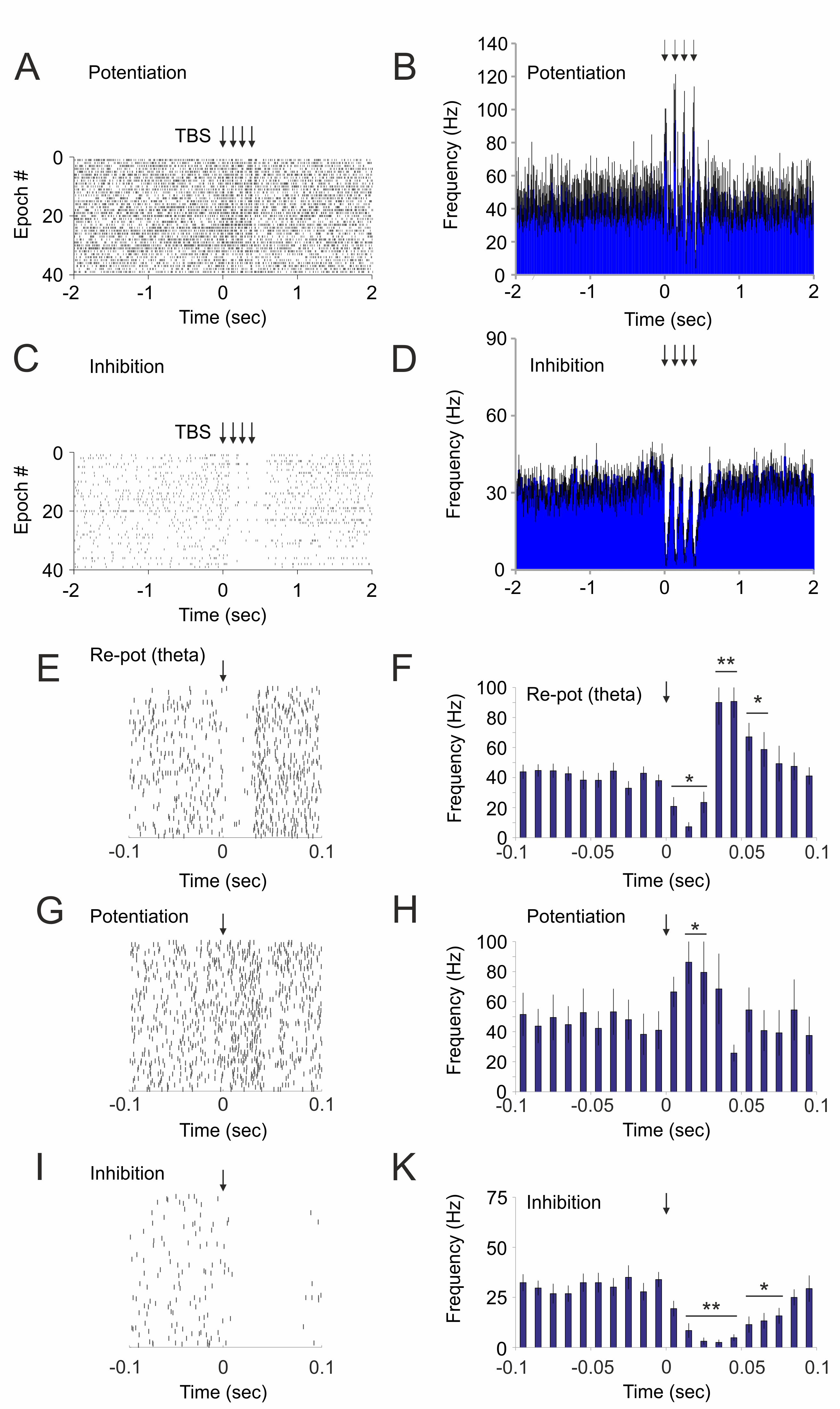

Supplement: Supplementary file 6 [file Image5.TIF]

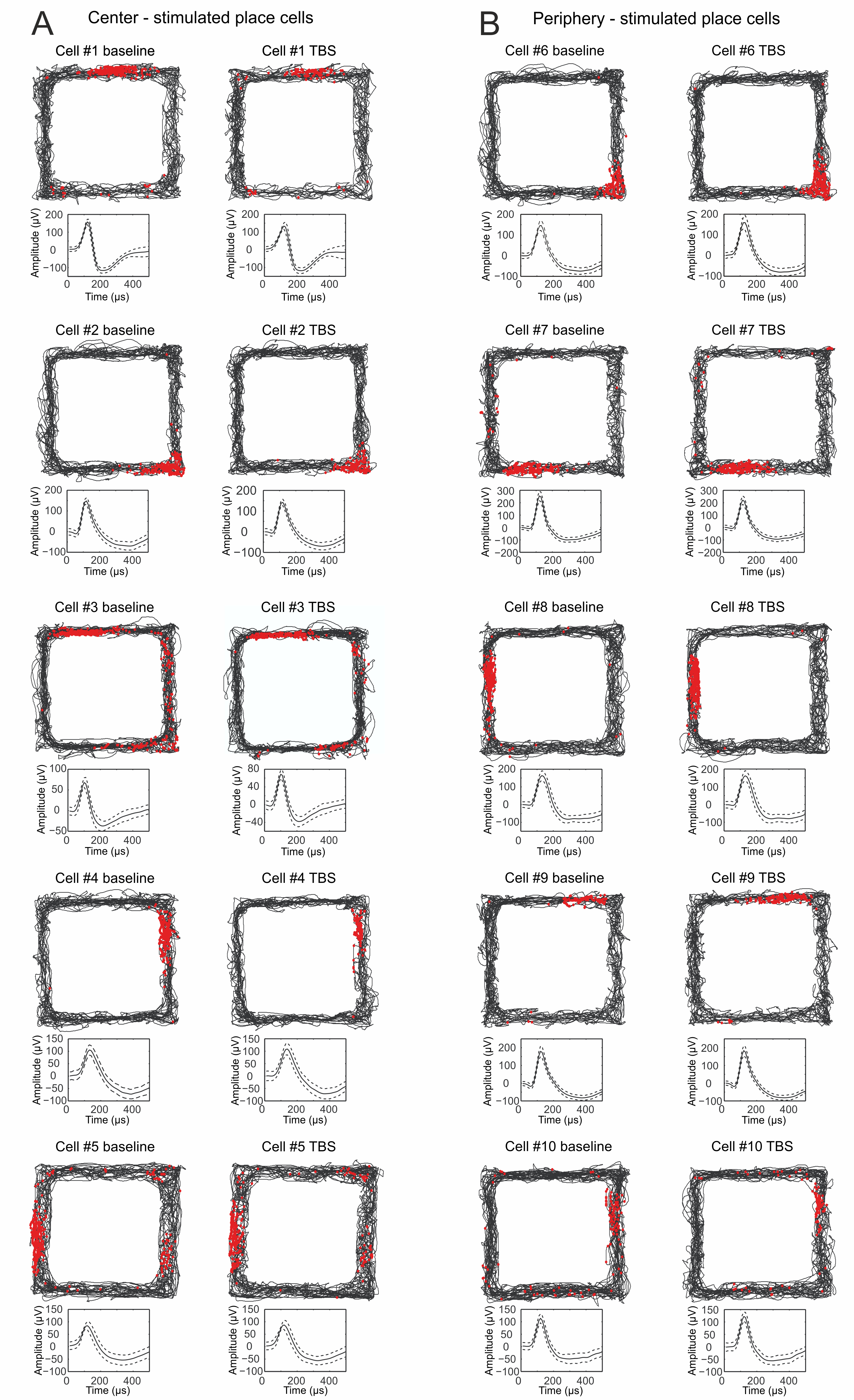

Supplement: Supplementary file 7 [file Image6.TIF]
